# Sex-specific role of high-fat diet and stress on behavior, energy metabolism, and the ventromedial hypothalamus

**DOI:** 10.1186/s13293-024-00628-w

**Published:** 2024-07-15

**Authors:** Sanutha Shetty, Samuel J. Duesman, Sanil Patel, Pacific Huynh, Pamela Toh, Sanjana Shroff, Anika Das, Disha Chowhan, Benjamin Keller, Johana Alvarez, Rachel Fisher-Foye, Robert Sebra, Kristin Beaumont, Cameron S. McAlpine, Prashant Rajbhandari, Abha K. Rajbhandari

**Affiliations:** 1 Department of Neuroscience and Psychiatry, Icahn School of Medicine at Mount Sinai, New York, NY 10029 USA; 2 Diabetes, Obesity and Metabolism Institute, Icahn School of Medicine at Mount Sinai, New York, NY 10029 USA; 3 Cardiovascular Research Institute, Icahn School of Medicine at Mount Sinai, New York, NY 10029 USA; 4 Center for Advanced Genomic Technology, Department of Genetics and Genomic Science, Icahn School of Medicine at Mount Sinai, New York, NY USA; 5 Center for Excellence in Youth Education, Icahn School of Medicine at Mount Sinai, New York, NY 10029 USA; 6 Disease Mechanism and Therapeutics Program, Icahn School of Medicine at Mount Sinai, New York, NY 10029 USA

## Abstract

**Background:**

Scientific evidence highlights the influence of biological sex on the relationship between stress and metabolic dysfunctions. However, there is limited understanding of how diet and stress concurrently contribute to metabolic dysregulation in both males and females. Our study aimed to investigate the combined effects of high-fat diet (HFD) induced obesity and repeated stress on fear-related behaviors, metabolic, immune, and hypothalamic outcomes in male and female mice.

**Methods:**

To investigate this, we used a highly reliable rodent behavioral model that faithfully recapitulates key aspects of post-traumatic stress disorder (PTSD)-like fear. We subjected mice to footshock stressor followed by a weekly singular footshock stressor or no stressor for 14 weeks while on either an HFD or chow diet. At weeks 10 and 14 we conducted glucose tolerance and insulin sensitivity measurements. Additionally, we placed the mice in metabolic chambers to perform indirect calorimetric measurements. Finally, we collected brain and peripheral tissues for cellular analysis.

**Results:**

We observed that HFD-induced obesity disrupted fear memory extinction, increased glucose intolerance, and affected energy expenditure specifically in male mice. Conversely, female mice on HFD exhibited reduced respiratory exchange ratio (RER), and a significant defect in glucose tolerance only when subjected to repeated stress. Furthermore, the combination of repeated stress and HFD led to sex-specific alterations in proinflammatory markers and hematopoietic stem cells across various peripheral metabolic tissues. Single-nuclei RNA sequencing (snRNAseq) analysis of the ventromedial hypothalamus (VMH) revealed microglial activation in female mice on HFD, while male mice on HFD exhibited astrocytic activation under repeated stress.

**Conclusions:**

Overall, our findings provide insights into complex interplay between repeated stress, high-fat diet regimen, and their cumulative effects on health, including their potential contribution to the development of PTSD-like stress and metabolic dysfunctions, emphasizing the need for further research to fully understand these interconnected pathways and their implications for health.

**Supplementary Information:**

The online version contains supplementary material available at 10.1186/s13293-024-00628-w.

## Plain Language Summary

In our study, we attempted to investigate how the combination of diet, stress, and sex can affect various aspects of health in mice. Specifically, we aimed to elucidate the neurobiology of underlying stress and metabolic dysfunction with a focus on sex-specific differences. We recognize that stress and metabolic disorders often co-occur and exhibit distinct patterns between sexes. In the present study, we observed that male mice fed a high-fat diet exhibited an inability to extinguish fear memory, mirroring a hallmark symptom observed in PTSD patients. We also observed sex-specific differences in metabolic and immune function in response to the diet and stress challenge. We uncovered that both repeated stress and a HFD can induce alterations in the quantity and types of immune cells present in various peripheral tissues, suggesting potential pathways through which metabolic diseases may develop. Our investigation further revealed that the ventromedial hypothalamus, responsible for regulating metabolism and stress behavior, exhibited distinct transcriptomic activity patterns in males and females. These findings shed light on the complex connections between high fat diet, stress levels, and overall health, emphasizing the importance of continued research in this area.

**Supplementary Information:**

The online version contains supplementary material available at 10.1186/s13293-024-00628-w.

## Highlights

Fourteen weeks of high fat diet (HFD) impaired fear memory extinction in stressed male mice but not in female mice.

Female mice demonstrated HFD-induced glucose intolerance only under repeated stress conditions.

Male mice on HFD showed an increase in energy expenditure and glucose intolerance, while female mice on HFD showed decreased respiratory exchange ratio (RER).

Furthermore, male mice on HFD showed decreased bone marrow progenitor cells, increased neutrophils in gonadal adipose tissue, decreased neutrophils in the aorta, and increased macrophages in the heart compared to female mice.

Single nuclei RNA sequencing (snRNAseq) analysis of the ventromedial hypothalamus revealed differential microglial and astrocytic cell activation in female and male mice respectively, with females showing elevated expression of *Hcrt* and *Pmch*, while males showing increased expression of *Gfap* and *Pomc*.

Together, these findings indicate distinct patterns in fear memory extinction, metabolic parameters, immune cell distribution, and gene expression within the ventromedial hypothalamus.

**Supplementary Information:**

The online version contains supplementary material available at 10.1186/s13293-024-00628-w.

## Background

Human and animal studies have consistently demonstrated that excessive consumption of high fat diet (HFD) leads to adverse health outcomes. These include weight gain [1, 2], glucose intolerance and insulin resistance [3, 4], and inflammation [5–7], all of which can lead to an elevated risk of chronic diseases such as diabetes [8], obesity [1, 9], and heart disease [10, 11]. Moreover, research suggests that HFD may exacerbate the body’s stress response by increasing circulating levels of stress hormones [12], notably cortisol [13–15]. This hormonal elevation can contribute to heightened stress levels and anxiety [16, 17]. Given the comorbidity of stress-related conditions like PTSD with metabolic diseases, it’s crucial to comprehensively understand how intense stressors interact with a high-fat diet (HFD). This knowledge is essential for uncovering the intricate relationships among stress, dietary habits, and their impact on overall health [18, 19].

Research investigating the influence of a HFD on cognitive flexibility in rats has uncovered that obese rats exhibit impaired cognitive flexibility, typically evaluated via a set-shifting task [20]. Memory impairments associated with a HFD have also been observed in a sex-specific manner. A study by Hwang et al. demonstrated that male mice fed a HFD for 12 weeks exhibited impaired learning performance in contextual fear conditioning [21]. Furthermore, studies have suggested that a HFD disrupts synaptic plasticity, particularly long-term potentiation (LTP), in the hippocampus, as observed in both mice and rats [21, 22]. These findings collectively indicate that a HFD can adversely impact stress response, cognitive function, memory deficits, and synaptic dysfunctions.

Additionally, studies indicate sex-specific differences in the response to HFD, particularly concerning weight gain and body composition [21, 23–25]. Decades of studies have used HFD-fed mice as models of diet-induced glucose intolerance and insulin resistance [3, 4, 8, 26, 27]. In the field of metabolism, glucose tolerance test (GTT) and insulin tolerance test (ITT) are commonly employed physiological assays to characterize dietary-induced type 2 diabetes-like phenotypes in rodents. These tests provide valuable insights into alterations in glucose metabolism by assessing the clearance of glucose load and insulin secretion over time [25]. Further, it is well-established that HFD-induced obesity triggers chronic low-grade inflammation, which can contribute to the development of metabolic disorders including insulin resistance and cardiovascular disorders [28–31]. Hence, the inflammatory response elicited under metabolic stress not only serves as biomarkers for cardiometabolic diseases but also actively contributes to the pathogenesis of these conditions [10, 11].

There are known sex-specific differences in stress responses, spanning from variations in freezing rates [32, 33] to shock reactivity [34]. These sexual dimorphisms could be attributed to differences in the hypothalamic-pituitary-adrenal (HPA) axis in rodents [35, 36]. Additionally, there is a recognized variability in how different brain regions like the hippocampus [37, 38], amygdala [39, 40], bed nucleus of the stria terminalis (BNST) [41], and arousal centers like locus coeruleus (LC) [42, 43], influence stress response in both male and female rodents. Of particular interest to us is the ventromedial hypothalamus (VMH), which is recognized for regulating fear and other emotional responses [44–46] in a sexually dimorphic manner. Additionally, the VMH is known to coordinate glucose and energy homeostasis in response to metabolic needs, also exhibiting sexual dimorphism [47–50]. As previously noted, the variations in stress responses are not isolated; it is crucial to consider additional environmental factors such as dietary influences.

While studies indicate that male mice may be more susceptible than females to the effects of a HFD on weight gain, metabolic changes, and cognitive function, the mechanisms at play in female mice could involve a more intricate and nuanced process [21, 23]. Stress-induced eating, often observed in individuals diagnosed with PTSD, particularly women, serves as a coping mechanism, increasing the risk of obesity and related health issues [51–53]. Despite this, there is limited understanding of how unhealthy dietary habits make an individual more susceptible to stress, affecting mood, fear regulation, and overall mental well-being. There hasn’t been a comprehensive study, including both sexes, investigating how diet and stress collectively impact fear behavior, metabolic health, immune functions, and the role of the VMH in coordinating these responses. This research is vital because clinical studies have revealed that symptoms of stress disorders such as PTSD impact metabolic outcomes. Therefore, it is imperative to comprehend the connection between diet and stress responses, especially within a sex-specific context.

Our study aimed to elucidate the intricate interactions between HFD and repeated footshock stress in male and female mice. We achieved this by investigating the effects of intense trauma-like footshock stressors combined with 14 weeks of HFD feeding, accompanied by weekly repeated reminder shocks. We conducted a thorough evaluation of the impact of acute and repeated stress, in conjunction with dietary factors, on behavior, body weight, energy metabolism, glucose homeostasis, food intake, immune cell enrichment in peripheral tissues, and the cellular state in the ventromedial hypothalamus.

## Materials and methods

### Animals

All the experimental procedures were conducted in accordance with the guidelines set by the Institutional Animal Care and Use Committee at the Icahn School of Medicine at Mount Sinai. A total of approximately 90 eight-week-old C57BL/6 mice, both males and females, were obtained from Jackson Laboratories. Mice were housed in a temperature-controlled room under a 12-h light–12-h dark cycle and under pathogen-free conditions. Mice were fed a chow diet (4.5% fat/calorie) except when indicated that the mice were fed a high-fat diet (60% fat/calorie). At the time of euthanization, tissues and blood were collected by cardiac puncture, frozen immediately, and stored at -80 °C.

### Experimental design

#### Diet/Repeated stress study

When the mice (both males and females) reached 10 weeks of age, they underwent stress-enhanced fear learning (SEFL) paradigm as described further in the subsequent sub-Sects. [43, 54–57]. Briefly, on day 1, the mice were placed in context A and received 10 randomized footshocks over 1 h. On day 2, they were placed in context B and received a reminder footshock 4 min after being in the context. Following an 8-minute SEFL test on day 3, the mice were divided into two groups: (i) a high-fat diet (HFD; 60% fat/calorie) group and (ii) a chow diet (4.5% fat/calorie) for 14 weeks. We chose this specific time frame because research indicates that metabolic alterations typically require prolonged feeding of a HFD for a minimum of 10 weeks [58, 59]. During this period, half of the mice in each group received weekly reminder shock (RS) in Context B of the SEFL, serving as a repeated stressor, while the remaining mice were placed in Context B without any shocks (no reminder shock; NRS) to allow for fear memory extinction.

At weeks 10 and 14 of the diet regimen, we conducted a glucose tolerance test (GTT) after 4-hour fast, administering a bolus of glucose to the mice and measuring their blood glucose levels over two hours. Subsequently, we placed all the mice in metabolic chambers for 72 h, as described in the methods section, for indirect calorimetric measurements of metabolic parameters. At week 14, we also conducted insulin tolerance tests (ITT), 4 days after the week 14 GTT test, to measure insulin sensitivity in response to an insulin bolus. Additionally, we assessed anxiety-like behaviors in the same mice by performing an open field light gradient behavioral task [60]. At the end of the experiments, the mice were euthanized, and tissues including brain, brown adipose tissue (BAT), gonadal white adipose tissue (gWAT), inguinal white adipose tissue (iWAT), liver, aorta, heart, bone marrow and blood were harvested for analysis.

#### Diet/Acute stress study

When the mice reached 10 weeks age, we divided the mice into HFD or chow groups and maintained them under the respective diets for 10 weeks. Following the 10-week dietary intervention, we subjected the mice to either acute stress or no stress, as described in our experimental methods below. Following this, we conducted GTT tests on the mice. Additionally, using the procedures detailed in the methods section, we utilized metabolic chambers to conduct indirect calorimetric measurements, allowing us to assess metabolic processes.

### Measure of freezing and shock reactivity

Freezing is a complete lack of movement except when the animal is breathing [54, 61]. Freezing was measured using EthoVision XT software, which uses real-time video recording. The software measures freezing via a method called activity detection. The software detects changes at the pixel level from one video frame to the next. The threshold for activity detection was set at 0.02%. Freezing was considered when the activity score remained below this threshold for 1 s. Percentage freezing = freezing time/total time×100 for the period of interest. The data are presented as the mean percentages (+/− SEM). Shock reactivity is an index of pain reactivity to the incoming foot shock. The activity burst velocity is a reliable predictor of foot-shock intensity and may be a useful indicator of pain reactivity. We calculate shock reactivity by the velocity of movement during the 1-s period during the shock on Day 2. The velocity in inches/s (+/− SEM) was computed for the period of interest.

### Stress-enhanced fear learning (SEFL)

SEFL is a robust and powerful rodent model that recapitulates many clinical aspects of PTSD-like fear [54, 62]. This model was established over a period of 3 days. On day 1, fear conditioning chambers were set up to serve as Context A in which the mice received the footshock stressors. For this, the mice were directly transported to the chambers from their home cages. Within the chamber, the conditional stimulus was presented in the form of white light, white noise, and the olfactory cue of a wet paper towel. All the mice were placed in the Context A for 1 h, and 10 random foot shocks of 1 mA were administered during this period. On day 2, we subjected all mice to a mild stressor (1 foot shock) in Context B and assessed their fear response to this stressor on day3. Context B serves as a safe context but the reminder footshock leads to memory of the stressors in Context A leading to enhanced fear responses. For this context, mice were transported into the behavior room individually in opaque containers. An extra light source was introduced on top of the chamber boxes, the walls of the chamber were altered to red, and the olfactory cue within the chamber was 1% acetic acid. However, the grid floors remained unchanged from day 1 to day 3. On day 2, the mice were placed in the chambers for a period of 4 min and 30 s. The mice received 1-foot shock of 1 mA at the end of the 4th minute to serve as a mild stressor. On day 3, the fear response to the mild stressor in context B was assessed by measuring freezing behavior. Mice were placed in the chambers for 8 min, and the percentage of freezing, defined as lack of movement, was calculated for the first 4 min of day 2 and 8 min of day 3. An additional measure of shock reactivity was calculated during the 4th-minute shock on day 2 using the same EthoVision XT system.

### Repeated stress

After SEFL, both HFD-fed and chow-treated male and female animals were randomized to undergo repeated stress (RS) or no repeated stress (NRS). The animals were placed week after week for 14 weeks in Context B for 4 min. We employed context B for the recurring stressor because it closely mirrors the “neutral/low stress” setting, where a mouse is not expected to exhibit a heightened fear response. This context aligns with our objective of observing the extinction of fear memories in NRS groups and a reinforcement of fear in RS groups. The RS group received one foot shock at the end of the 4th minute, while the individuals in the NRS groups were allowed to extinguish the fear memory that developed through the SEFL.

### Fear test

At the end of the 14-week repeated stress paradigm, we conducted an 8-minute fear test in Context B. In this test, the animals were placed in Context B (with same mode of transportation and cues) to test their freezing response in the absence of any foot shocks for both RS and NRS groups. This test is like the SEFL Day 3 test.

### Glucose tolerance test

For the glucose tolerance test, after 10 and 14 weeks of feeding with either a HFD or chow in both experimental groups, the mice were fasted for 4 h. The baseline blood glucose levels (mg/dL) of these mice were measured using one drop of blood collected from the tip of the tail and a glucometer. For the test, 1 g of glucose/kg body weight was injected intraperitoneally (i.p.) into each mouse. Blood glucose levels were determined at 0, 15, 30, 60, 90, and 120 min.

### Indirect calorimetry using a metabolic chamber

The mice were placed in an indirect calorimetry chamber for 72 h to study metabolic function after 10 weeks of diet + trauma administration in acute stress and repeated stress experiments. The data collected via indirect calorimetry included oxygen consumption, carbon dioxide production, energy expenditure (EE), the respiratory exchange ratio (RER), energy balance, food and water intake, locomotor activity, and body mass. For some indirect calorimetry measurements in the metabolic chambers, such as oxygen consumption, carbon dioxide production, EE, and food and water intake, body weight was considered a covariate in the statistical analysis. The RER and locomotion were analyzed without a covariate, as they are known to be independent of body weight. This information highlights changes in metabolic physiology in HFD/Chow diet-fed animals after receiving either RS/ NRS or acute stress/ no stress. We analyzed the data with CalR [63], which considers activity, food intake, and other parameters, allowing us to derive accurate indirect calorimetry values.

### Insulin tolerance test

For the insulin tolerance test (ITT), mice were maintained on a HFD or chow diet for 10 weeks and were fasted for 4 h after 14 weeks before receiving an i.p. injection of recombinant human insulin (1 U/kg body weight). Blood glucose levels were determined at 0, 15, 30, 60, 90, and 120 min using a glucometer.

### Open field light gradient anxiety test

The mice were also tested for anxiety-like behavior in a modified open-field task that incorporated a light gradient [60]. The open field light gradient test was used to measure anxiety-like phenotypes through the use of parameters such as distance traveled, velocity, and time spent in the dark zone. A rectangular, white, translucent polyethylene box (46 × 86 × 30 cm) was placed in a dark room. Three lamps were placed on one end of the box. A camera suspended above captured the animal’s activity. The test was divided into three phases during the 12-minute run. During the first four minutes, the lights are turned off, and the animal can explore the open field in the dark phase 1. During the next four minutes, the lamps are turned on, and a light gradient is created in the open field. The zone closest to the light source, called the “light zone”, has an illumination index of ~ 1200 lx. The “middle zone” has intermediate illumination of ~ 50 lx, and the zone farthest from the light source, the “dark zone”, has an illumination index of ~ 10 lx. In the last phase of the experiment, dark phase 2, the lights are turned off again. The locomotive activity, velocity, and time spent in the dark zone were calculated using EthoVision XT software.

### Acute stress

We used a two-day acute stress model in mice to study the effect of diet and acute stress like the protocol followed by Duesman et al. [43]. Mice that were fed either chow or a HFD either received acute stress (10 randomized foot shocks over 1 h) or no stress. On day 1, the mice were placed in the fear conditioning chamber Context (A) In context A, the mice were directly transported to the chambers from their home cages. Within the chamber, the conditional stimulus was presented in the form of white light, white noise, or the olfactory cue of a wet paper towel. The mice were placed in this chamber for a period of 1 h, where the group of mice that were receiving acute stress was presented with 10 random foot shocks of 1 mA. The control animals in both dietary groups did not receive any foot shocks and are referred to as the no-stress group. Fear generalization was tested on day 2 in context (B) In this context, the mode of transport and the light, visual, and olfactory cues used were drastically different. Mice were subsequently transported into the behavior room in individual opaque containers. An additional source of light was added on top of the chamber boxes, the walls of the chamber were changed to red, and the olfactory cue within the chamber was now 1% acetic acid. The grid floors are, however, kept the same from day 1 to day 2. The mice were placed in this new context for a period of 4 min, during which their fear was measured as an index of freezing.

### Tissue harvests

After completing all the above experiments, the mice were food deprived for 4 h, anesthetized with isoflurane, and rapidly decapitated. Multiple tissues were harvested and rapidly stored in a -80 °C freezer. iWAT, gWAT, BAT, blood, liver, and brain tissues were harvested from the acute stress groups. In addition to these tissues, the heart, aorta, and bone marrow were harvested from the RS groups.

### Cell collection

Blood was collected, and RBC lysis buffer (BioLegend) was used twice to lyse the red blood cells. After transcardiac perfusion with PBS (Thermo Fisher Scientific), organs (infarcted cardiac tissue and brain tissue) were collected, minced, and digested in a mixture of 450 U/mL collagenase I, 125 U/mL collagenase XI, 60 U/mL DNase and 60 U/mL hyaluronidase (Sigma‒Aldrich) in PBS for 45 h on a shaker (at 800 rpm) at 37 °C. Next, the digested organ was flushed through a 100 μm cell strainer. The brain suspensions were further purified using a Percoll density gradient (30% upper and 70% lower phase). The cell layer between the two phases was collected and washed in PBS. Bone marrow cells were flushed from the bone marrow cavities and resuspended in a single-cell suspension by pipetting up and down; then, RBC lysis buffer was used to lyse the red blood cells.

### Flow cytometry

Single-cell suspensions were stained in FACS buffer (0.5% BSA and 2 mM EDTA in PBS) containing fluorophore-coupled antibodies at a concentration of 1:700 at 4 °C for 30 min, unless otherwise indicated. To differentiate between live and dead cells, the cell suspensions were stained with Live/Dead Blue (ThermoFisher) at a concentration of 1:1,000 in PBS at 4 °C for 30 min. The following antibodies were used for flow cytometry: anti-CD45 (BioLegend, 30-F11), anti-CD11b (BioLegend, M1/70), anti-CD90.2 (Invitrogen, 53 − 2.1 and BioLegend, 30-H12), anti-B220 (BioLegend, RA3-6B2), anti-CD19 (BioLegend, 6D5), anti-CX3CR1 (BioLegend, SA011F11), anti-Ly-6G (BioLegend, 1A8), anti-Ly-6 C (BioLegend, HK1.4), anti-f4/80 (BioLegend, BM8), anti-MHCII (BioLegend, M5/114.15.2), anti-CD64 (BioLegend, X54-5/7.1), anti-CD49b (BioLegend, DX5), anti-Ter119 (BioLegend, TER-119), anti-CD11c (BioLegend, N418), anti-CD127 (BioLegend, S18006K), anti-cKit (BioLegend, 2B8), anti-Sca-1 (BioLegend, E13-161.7), anti-CD135 (BioLegend, A2F10), anti-CD48 (BioLegend, HM48-1), anti- CD150 (BioLegend, TC15-12F12.2), anti-CD34 (eBioscience, RAM34), anti-CD16/32 (BioLegend, 93), anti-CD115 (BioLegend, AFS98), anti-BrdU (eBioscience, BU20A). The following primary antibodies were used: anti-feeder (Miltenyi Biotec, mEF-SK4), anti-podoplanin/gp38 (BioLegend, 8.1.1), and anti-CD31 (BioLegend, 390). Mature cells were identified as follows: (1) Ly6CHi monocytes (CD45 + CD11b + CX3CR1 + f4/80- Ly6CHi), (2) neutrophils (CD45 + CD11b + CX3CR1-Ly6G), (3) macrophages (CD45 + CD11b + CX3CR1 + f4/80 + CD64+), (4) fibroblasts (CD45-CD31-gp38 + mEFSK4+), (5) T cells (CD45 + CD11b-CD90.2+), (6) B cells (CD45 + CD11b-B220+), and (7) microglia (CD45 + midCD11b+). The following progenitor cells were identified: (1) LSK (CD45 + Lin-Sca1 + cKit+), (2) granulocyte-macrophage progenitor (CD45 + Lin − cKit + Sca1 − CD34 + CD16/32HiCD115−), (3) monocyte-dendritic cell progenitor (CD45 + Lin − cKit + Sca1 − CD34 + CD16/32HiCD115+), and (4) common myeloid progenitor (CD45 + Lin − cKit + Sca1 − CD34-CD16/32mid). The lineage was identified as Lin: B220, CD19, CD49b, Ter119, CD90.2, CD11b, CD11c, Ly6G, and CD127. The data were acquired using a Cytek Aurora (Cytek) and analyzed with FlowJo (Tree Star).

### RNA purification, cDNA synthesis, and RT‒qPCR

RNA was isolated from brown adipose tissue (BAT) and liver tissue using phenol–chloroform extraction. After isolation, the RNA pellet was washed and resuspended in diethylpyrocarbonate (DEPC) water at a concentration of 200 ng/µL. The RNA samples were reverse transcribed to cDNA using a high-capacity cDNA reverse transcription kit (Applied Biosystems). Real-time qPCR was performed by using real-time PCR with SYBR Green master mix (Diagenode). Samples were run and analyzed on a Quantstudio 5 (Applied Biosystems). The qPCR data were normalized to the expression of the housekeeping gene 36B4.

### Single-nuclei RNA sequencing (snRNA-seq)

Starting with the fresh-frozen VMH, approximately 100 mg of tissue was obtained by cutting brain punches, and the VMH regions from five mice were combined. Single-nucleus gene expression sequencing was performed on the samples using the Chromium platform (10x Genomics, Pleasanton, CA) with the Next GEM Single-cell 3’GEX Reagent Kit. Briefly, nuclei were isolated from frozen tissue using a Singulator instrument (S2 Genomics, Livermore, CA) via the “Low Input Nuclei Isolation” protocol, where the mixture was modified to the “top” type and “slow” speed. Additionally, disruption was performed via the “Dounce” method at “Medium” speed. Once run, 3–4 mL of the recovered supernatant was centrifuged at 300 × g for 4 min at 4 °C and resuspended in 100 µL of PBS + 0.04% BSA, followed by filtration using a 40 μm cell strainer. Following the loading of ~ 10,000 healthy nuclei, Gel-Bead in Emulsion (GEMs) were generated on the sample chip in the Chromium controller. Barcoded cDNA was extracted from the GEMs by post-GEM RT-cleanup and amplified for 12 cycles. Amplified cDNA was fragmented and subjected to end repair, poly A-tailing, adapter ligation, and 10X-specific sample indexing following the manufacturer’s protocol. Libraries were quantified using a bioanalyzer (Agilent) and QuBit (Thermo Fisher) analysis. Libraries were sequenced using a 2 × 100PE configuration on a NovaSeq instrument (Illumina, San Diego, CA) targeting a depth of 50,000-100,000 reads per nucleus. Sequencing data were aligned and quantified using the Cell Ranger Single-Cell Software Suite (version 7.1.0, 10x Genomics) against the provided mm10 reference genome using default parameters, including introns.

### snRNA-seq preprocessing and quality control

To obtain digital gene expression matrices (DEGs) with sparse matrix representation, paired-end reads from the Illumina NOVA-seq platform were processed and mapped to the mm10 mouse genome using the 10X Genomics Cell Ranger v3.0.2 software suite. Briefly,.bcl files from the Mount Sinai sequencing core were demultiplexed and converted to fastq format using the ‘mkfastq’ function from Cell Ranger. Next, the Cell Ranger ‘counts’ function mapped reads from fastq files to the mm10 reference genome and tagged mapped reads as exonic, intronic, or intergenic. Only reads that were aligned to exonic regions were used in the resulting DEGs. After combining all four sample DGEs into a single study DGE, we filtered out cells with (1) UMI counts < 700 or > 30,000, (2) gene counts < 200 or > 8,000, or (3) a mitochondrial gene ratio > 10%. This filtering resulted in a dataset consisting of approximately 2,300–4,650 cells from each sample. A median of 2,411 genes and 7,252 transcripts were detected per cell.

### Identification of cell clusters

To achieve high-resolution cell type identification and increased confidence in our cell type clustering, we collected external publicly available data. The single-cell expression profiles were projected into two dimensions using the UMAP method [64] for community detection. These integrated data were only used to identify and define the cell types. All plots that were not explicitly designated as integrated with at least one external dataset and all downstream analyses (e.g., differential expression analyses) were constructed on nonintegrated data to retain the biological effect of the cold treatment. Visualization of the nonintegrated data was conducted on a subsampled dataset where all samples had the same number of cells to give equal weight to each sample; however, all downstream analyses (e.g., differential expression analyses) leveraged the full dataset.

### Cell type-specific marker gene signatures

Cell type-specific marker gene signatures were generated by identifying genes whose expression levels were twofold greater (adjusted p values < 0.05) than those of all other cell types. To ensure consistency across samples, Seurat’s FindConservedMarkers function (Wilcoxon rank sum test with a meta p value) was applied across each sample.

### Resolving cell identities of the cell clusters

To determine the cell type identity of each cluster, we used a curated set of canonical marker genes derived from the literature to find distinct expression patterns in the cell clusters. Clusters that uniquely expressed known marker genes were used as evidence to identify that cell type. Cell subtypes that did not express previously established markers were labeled with both general cell type markers and novel markers obtained with Seurat’s FindConservedMarkers function and were used to define the cell subtype.

### Differential gene expression analysis

Within each identified cell type or subtype, cold-treated and room temperature single cells were compared for differential gene expression using Seurat’s FindMarkers function (Wilcoxon rank sum test) in a manner like that used by Li et al. [65]. DEGs were identified using two criteria: (i) an expression difference > = 1.5-fold and adjusted p value < 0.05 in a grouped analysis between room temperature mice (*n* = 2) and cold-treated mice (*n* = 2) and (ii) an expression difference > = 1.25-fold and consistent fold change direction in all 4 possible pairwise combinations of cold-treated vs. room temperature mice.

#### Gene regulatory network inference

Gene regulatory network inference was performed with pySCENIC following the workflow described by Van de Sande et al. [66]. Briefly, starting with count data, gene modules that were coexpressed with transcription factors were identified with GRNBoost2 [67]. Next, candidate regulons were created from transcription factor–target gene interactions, and indirect targets were pruned based on motif discovery with cisTarget [66]. Finally, regulon activity was quantified at cellular resolution with AUCell [66], which allowed for the prioritization of regulons for each cell type based on the quantified activity.

### S**tatistical analysis**

The data are shown as the mean ± S.E.M. The distribution of the proteins was assessed by the Shapiro‒Wilk test. Significance was determined by a two-tailed unpaired t test (parametric distribution), 2-way ANOVA with the Bonferroni *post hoc* correction, ANCOVA, Tukey’s multiple comparison test, Sidak’s multiple comparison test, or the Mann‒Whitney test (nonparametric distribution). The significance level was set at an alpha level of 0.05.

## Results

### HFD led to impaired fear extinction in male but not female mice

**Fig. 1:**
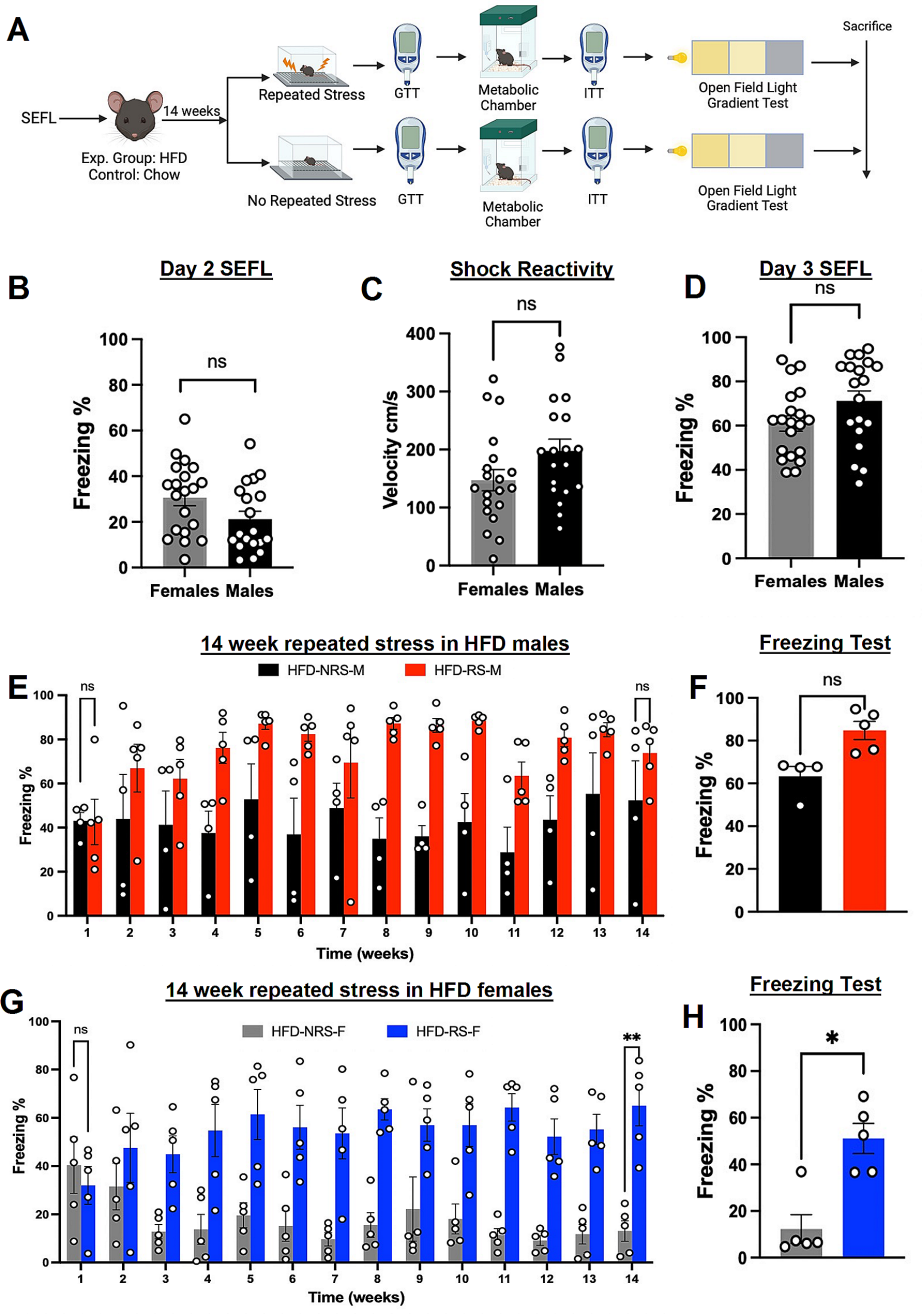
A high-fat diet inhibits fear extinction in male mice. **(A)** Experimental timeline used to study the effect of diet and repeated stress on mice. **(B)** Percent freezing on day 2 of SEFL (*n* = 20/19; two-tailed unpaired t test, t = 1.913; *p* = 0.0635). **(C)** Shock reactivity measured as velocity (cm/s) on day 2 (*n* = 20/19; two-tailed unpaired t test, t = 1.875; *p* = 0.0687). **(D)** Percent freezing on day 3 of SEFL (*n* = 20/19; two-tailed unpaired t test, t = 1.814; *p* = 0.3916). **(E)** Percent freezing of HFD-fed male mice during 14 weeks of RS/NRS treatment (*n* = 4,5; two-way ANOVA, F_1,98_ =69.18; ****p < 0.0001; *post hoc* comparison, *n* = 5/4; Sidak’s multiple comparisons test, *p* > 0.99). **(F)** The freezing percentage of HFD-fed male mice during the freezing test conducted after 14 weeks (*n* = 5/4, two-tailed paired t test; t = 2.518, *p* = 0.08). **(G)** Percent freezing of HFD female mice during 14 weeks of RS/NRS treatment (*n* = 5; two-way ANOVA, F_1,112_ =157.1; ****p < 0.0001) (*post hoc* comparison, *n* = 5; Sidak’s multiple comparisons test, **p < 0.01). **(H)** Percent freezing in HFD females in the freezing test after 14 weeks (*n* = 5, two-tailed paired t test; t = 3.514, *p < 0.05)

The study’s experimental design is illustrated in Fig. 1A. First, we assessed the percentage of freezing behavior of female and male mice during SEFL paradigm before the dietary regimen. To determine the possibility of fear generalization on Day 2 of SEFL, we assessed percentage of freezing behavior in female and male mice. There were no observed differences between the groups (Fig. 1B) (*n* = 19,20; two-tailed unpaired t test, t = 1.913, *p* = 0.0635). We also observed no difference in shock reactivity between the groups to the foot shock delivered in context B on day 2 (Fig. 1C) (*n* = 19,20; two-tailed unpaired t test, t = 1.875, *p* = 0.0687). Our study revealed no statistically significant differences between female and male mice in terms of the percentage of freezing on day 3 of SEFL paradigm before the dietary regimen (Fig. 1D) (*n* = 19,20; two-tailed unpaired t test, t = 1.814, *p* = 0.0774). However, during the 14-week period, we found a main effect of stress on HFD-fed male mice that were subjected to repeated shocks (Fig. 1E *n* = 4,5; two-way ANOVA; F_1,98_ =69.18, *p* < 0.05). Post hoc comparisons revealed that by week 14, there were no significant differences in freezing behavior between male mice subjected to HFD-NRS or HFD-RS (*n* = 4,5; Sidak’s multiple comparison test; *p* > 0.99) (Fig. 1E). These findings indicate that male mice fed a HFD did not exhibit fear extinction by week 14, as evidenced by the consistently high percentage of freezing. This persistent response was notably also not found to be statistically different from HFD-RS male mice during the freezing test conducted after the 14-week period (Fig. 1F) (*n* = 4,5; two-tailed paired t test; t = 2.518, *p* = 0.0864). Two-way ANOVA of HFD-fed female mice revealed a main effect of stress (*n* = 5; two-way ANOVA, F_1,112_ =157.1, *p* < 0.05). Post hoc comparisons revealed a significant reduction in freezing percentage in the NRS group compared to that in the RS group at week 14 (Fig. 1G) (*n* = 5; Sidak’s multiple comparison test; *p* < 0.05). Similarly, NRS-female mice exhibited a significant reduction in % freezing during the freezing test conducted at week 14 (Fig. 1H) (*n* = 5; two-tailed paired t test; t = 3.514, *p* < 0.05).

In the chow fed male and female mice groups, we observed a reduction in % freezing by week 14 (*n* = 5 males; two-way ANOVA; F_1,112_ =89.76, *p* < 0.05) (*n* = 5 males, Sidak’s multiple comparison test, *p* < 0.0001) (*n* = 5 females, two-way ANOVA; F_1,112_ =161.1, *p* < 0.05) (*n* = 5 females, Sidak’s multiple comparison test; *p* < 0.0001). (Sup. Figure S1 A & S1 C) in the chow-NRS group compared to that in the chow-RS groups. Additionally, we observed a significant decrease in the percentage of freezing behavior during the freezing test conducted at week 14 in both male and female mice (Sup. Figure S1B & S1D) (*n* = 5 males, two-tailed paired t test; t = 3.586, *p* = 0.023). (*n* = 5 females, two-tailed paired t test; t = 4.771, *p* = 0.008). In the open field light gradient tests conducted after, there were no main effects of diet or sex in any of the HFD/chow groups (*n* = 4,5, two-way ANOVA; *p* > 0.05) (Supplemental Fig. 1E-1G).

### HFD and repeated stress induced weight gain and disrupted blood glucose homeostasis in both male and female mice

**Fig. 2:**
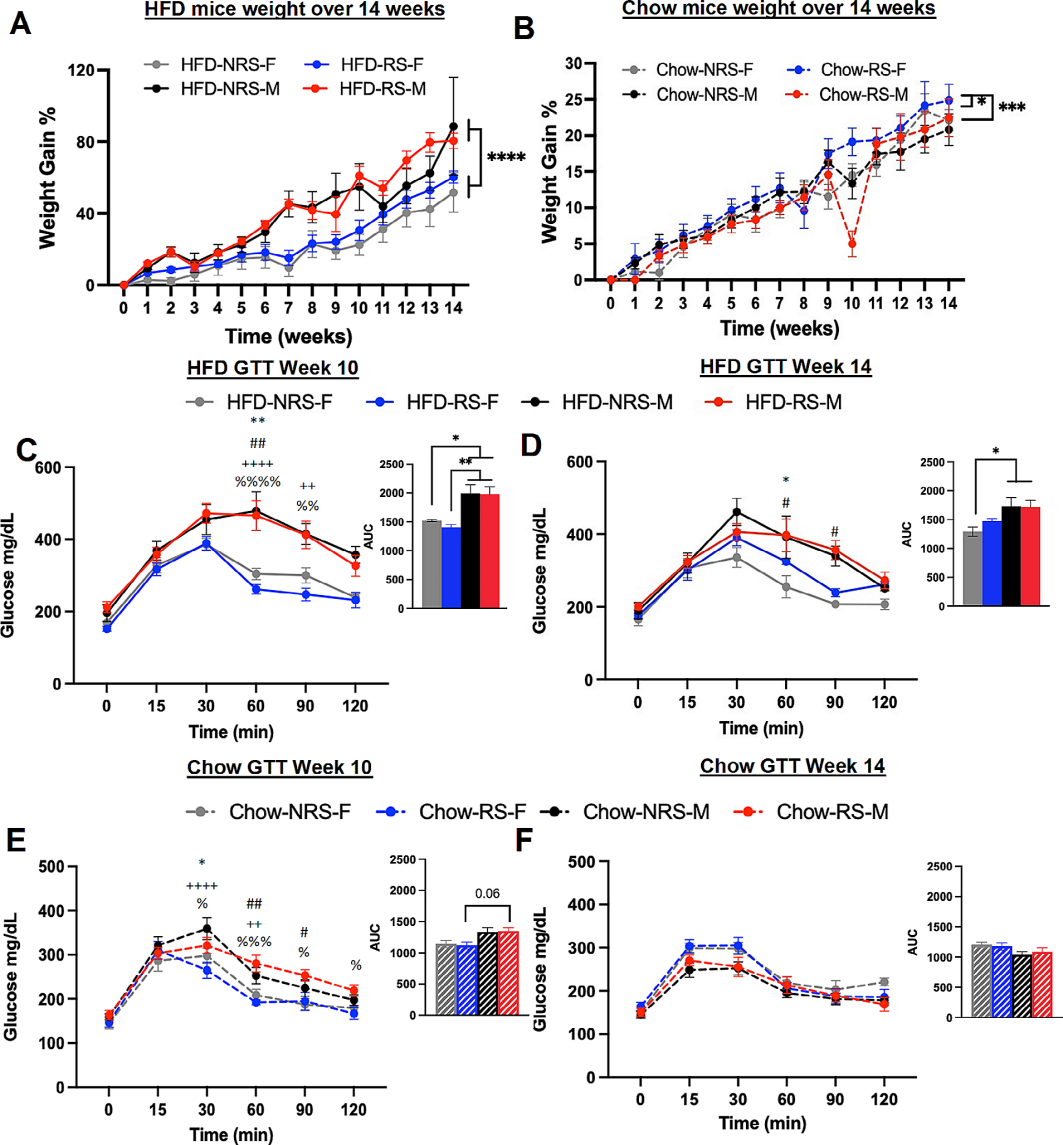
HFD induces weight gain and an increase in blood glucose levels in a sex-specific manner. **(A)** Percent weight gain over 14 weeks in HFD-fed mice (*n* = 4/5, two-way ANOVA; F _3,225_ =37.12; ****p < 0.0001) (*Post hoc* comparison, *n* = 4/5, Tukey’s multiple comparisons test, *p* < 0.05 from week 7). **(B)** Percent weight gain over 14 weeks plotted for chow-fed mice (*n* = 5; two-way ANOVA, F _3,240_ =5.698, ***p < 0.001) (*Post hoc* comparison, *n* = 5, Tukey’s multiple comparisons test, *p* < 0.05 in week 10 only). **(C)** Week 10 plasma glucose levels during the GTT test after 4 h of fasting in HFD mice (*n* = 5, two-way ANOVA, F _3,90_=33.23, ****p < 0.0001) (*post hoc* comparison, *n* = 5, Tukey’s multiple comparisons test, *p* < 0.05). AUC graph from the 10-week GTT test in HFD mice (*N* = 5, one-way ANOVA, F _3,15_ =1.579, ***p < 0.001) (*post hoc* comparison, *N* = 5, Tukey’s multiple comparisons test, *p* < 0.05) **(D)** Week 14 plasma glucose levels during the GTT test in HFD mice (*n* = 5, two-way ANOVA, F _3,90_=14.91, ****p < 0.0001) (*post hoc* comparison, *N* = 5, Tukey’s multiple comparisons test, *p* < 0.05). AUC graph from the 14-week GTT test in HFD mice (*n* = 5, one-way ANOVA; F _3,15_=0.8293, *p < 0.05) (*post hoc* comparison, *n* = 5; Tukey’s multiple comparison test; *p* < 0.05). **(E)** Week 10 plasma glucose levels during the GTT test in chow-fed mice (*n* = 5, two-way ANOVA, F _3,96_=12.54, ****p < 0.0001) (*post hoc* comparison, *n* = 4/5, Tukey’s multiple comparisons tests, *p* < 0.05). AUC graph from the 10-week GTT test in chow-fed mice (*n* = 5, one-way ANOVA; F _3,16_=0.0884, *p < 0.05) (*post hoc* comparison, *n* = 4/5, Tukey’s multiple comparisons test, *p* < 0.05). **(F)** Week 14 plasma glucose levels during the GTT test in chow-fed mice (*n* = 5, two-way ANOVA, F _3,96_=6.201, ***p < 0.001) (*post hoc* comparison, *n* = 4/5, Tukey’s multiple comparisons tests, *p* = 0.08). AUC graph from the 14-week GTT test in chow-fed mice (*n* = 5, one-way ANOVA; F _3,65_=0.075; *p* = 0.59) (*Compares No repeated Shock F vs. No repeated Shock M, #Compares No repeated shock F to Repeated Shock M, +Compares Repeated shock F to No repeated Shock M, %Compares Repeated shock F to Repeated Shock M)

We observed that male and female mice were significantly different in percentage of weight gain under HFD (*n* = 4,5; two-way ANOVA; F _32,235_ =37.12, *p* < 0.0001) (Fig. 2A). *Post hoc* comparisons revealed a statistically significant difference in weight gain between male and female mice within the HFD-fed groups (*n* = 4,5; Tukey’s multiple comparison test; *p* < 0.0001). Similarly, as seen in Fig. 2B, male and female mice fed a Chow diet displayed a significant differences in % weight gain with males increasing weight at a higher rate than females (*n* = 5; two-way ANOVA; F _3,240_ =5.698, *p* = 0.009). *Post hoc* comparison revealed a significant difference between chow-RS-F and chow-NRS-F and between chow-RS-F and chow-RS-M (*n* = 5, Tukey’s multiple comparison test, *p* = 0.0097). Next, we examined whether repeated stress and a HFD influenced glucose tolerance and insulin sensitivity through a glucose tolerance test (GTT) and an insulin tolerance test (ITT), respectively. Our results revealed glucose levels were significantly different in male mice and female mice fed HFD (Fig. 2C) (*n* = 5; two-way ANOVA; F _3,90_ =33.23, *p* < 0.0001). *Post hoc* analysis revealed that the differences were significant at the 60- to 90-minute time points in the HFD-female group compared with the HFD-male group (*n* = 5; Tukey’s multiple comparison test; *p* < 0.05, *p* < 0.01, *p* < 0.001, *p* < 0.0001). Furthermore, the area under the curve (AUC) plot also revealed differences between male and female mice (Fig. 2D) (*n* = 5; one-way ANOVA; F _3,15_ =9.531, *p* = 0.0009). *Post hoc* comparison revealed differences between male and females fed HFD irrespective of stress grouping (*n* = 4,5, Tukey’s multiple comparison test, *p* < 0.05, *p* < 0.01). By week 14, we also observed a significant effect of sex and stress on blood glucose levels (*n* = 5, two-way ANOVA; F _3,90_ =14.91, *p* < 0.0001). *Post hoc* analysis confirmed a significantly elevated blood glucose level in male mice in the HFD-RS and HFD-NRS group, particularly at the 30- to 90-minute time points, compared to that in the HFD-NRS female groups (*n* = 5; Tukey’s multiple comparison tests; *p* < 0.05). Subsequently, female mice in the HFD-RS group exhibited heightened blood glucose levels and no longer showed blunted glucose levels in comparison to HFD-male mice. Further, we observe a sex effect in the AUC graph (*n* = 5; one-way ANOVA; F _3,15_ =4.605, *p* = 0.017) (Fig. 2D). *Post hoc* comparison shows significant differences between HFD-NRS-F and both HFD male groups (*n* = 4,5, Tukey’s multiple comparison test, *p* = 0.03). Subsequently, we assessed blood glucose levels at 10 and 14 weeks in male and female mice from the chow groups in the NRS and RS. At week 10, we observed a distinct effect of sex and stress on blood glucose levels in chow fed mice (Fig. 2E) (*n* = 4,5; two-way ANOVA; F _3,90_ =49.41, *p* < 0.0001). *Post hoc* analysis indicated that the differences started to increase at the 30-minute time point and were sustained until the 120th minute of measurement (*n* = 4,5; Tukey’s multiple comparison tests; *p* < 0.05, *p* < 0.01, *p* < 0.001, *p* < 0.0001). The area under the curve (AUC) further showed a significant sex and stress effect (*n* = 4,5; one-way ANOVA; F _3,16_ =4.288, *p* = 0.0212). At week 14, however, we found no significant effect of the sex or stress on the GTT in male or female mice fed chow (Fig. 2F) (*n* = 4,5; two-way ANOVA; F _3,96_ =59.52, *p* = 0.618). The area under the curve (AUC) graph further illustrates no difference between the groups (*n* = 4,5; one-way ANOVA; F _3,16_ =1.867, *p* = 0.1758).

We conducted ITTs on 14-week-old HFD-fed, chow-fed female and male mice subjected to either NRS or RS. We did not see any effect of stress or sex in HFD fed mice (Sup. Figure 2 A) (*n* = 5; two-way ANOVA; F _3,16_ =1.438, *p* = 0.38) further confirmed by the area under the curve (AUC) (*n* = 5; one-way ANOVA; *p* = 0.595). However, in chow-fed mice, we see a significant effect of sex (Sup. Figure 2B) (*n* = 4,5; two-way ANOVA; F _3,96_ =112.1, *p* < 0.0001). *Post hoc* analysis revealed that, compared with chow-fed female mice, chow-fed male mice exhibited higher blood glucose revealing higher insulin sensitivity at all time points (*n* = 4,5; Tukey’s multiple comparison tests, *p* < 0.05, *p* < 0.01, *p* < 0.001, *p* < 0.0001). The area under the curve (AUC) further confirmed the observed differences in ITTs between the diet groups of male mice (*n* = 5; one-way ANOVA; F _3,16_ =68.94, *p* < 0.0001).

### Differential effects of HFD and repeated stress on energy balance

**Fig. 3:**
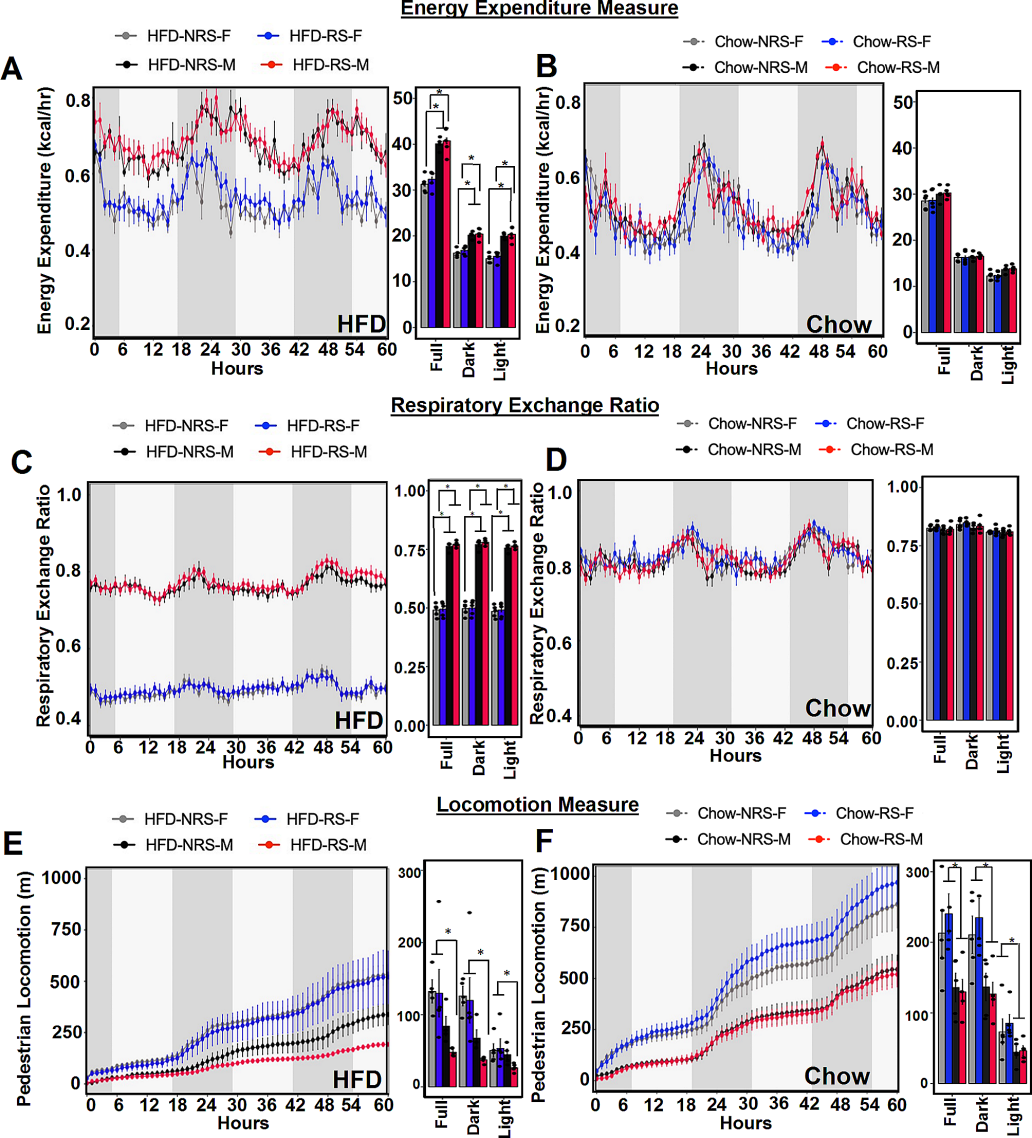
A HFD and repeated reminder shocks led to sex-specific metabolic dysregulation. **(A)** Energy expenditure (kCal/hr) of HFD-fed mice over 60 h in the metabolic chamber (*n* = 4,5; one-way ANOVA, *p* < 0.05) (*post hoc* comparison, *n* = 4,5; Tukey’s multiple comparisons test, *p < 0.0**5**). **(B)** Energy expenditure (kCal/hr) of chow-fed mice over 60 h in the metabolic chamber (*n* = 5; one-way ANOVA, *p* > 0.05). **(C)** Respiratory exchange ratio (RER) of HFD-fed mice aged more than 60 h in the metabolic chamber (*n* = 4/5; one-way ANOVA, *p* < 0.05) (*post hoc* comparisons, *n* = 4,5; Tukey’s multiple comparisons test, *p < 0.05). **(D)** RERs of chow-fed mice aged more than 60 h in the metabolic chamber (*n* = 5; one-way ANOVA, *p* > 0.05). **(E)** Pedestrian locomotion in HFD-fed mice over 60 h in the metabolic chamber (*n* = 5; one-way ANOVA, *p* < 0.05) (*post hoc* comparisons, *n* = 4,5; Tukey’s multiple comparison test, *p < 0.05). **(F)** Pedestrian locomotion in chow-fed mice over 60 h in the metabolic chamber (*n* = 5; one-way ANOVA, *p* < 0.05) (*post hoc* comparison, *n* = 4,5, Tukey’s multiple comparisons test, *p < 0.05)

To explore the impact of diet and repeated shocks on energy metabolism, we subjected both chow-fed and HFD-fed mice from both the RS and NRS groups to a 72-hour period in metabolic chambers. This approach allowed us to measure indirect calorimetry parameters, including energy expenditure (EE), the respiratory exchange ratio (RER), food intake, and locomotion. In our analysis, we excluded the initial 12 h to allow the mice to acclimatize. We observed a main effect of sex on the energy expenditure of mice fed a HFD (Fig. 3A) (*n* = 4,5; one-way ANOVA; *p* < 0.05). *Post hoc* comparison revealed that under HFD conditions, male mice exhibited significantly greater EE than female mice. However, EE was comparable between the RS and NRS groups within both male and female mice, indicating that there was no discernible stress effect on EE (*n* = 4,5; Tukey’s multiple comparison test; *p* < 0.05). In the context of a chow diet, there was no discernible impact of sex or stress on overall EE (Fig. 3B) (*n* = 5; one-way ANOVA; *p* > 0.05). Subsequently, we investigated fuel utilization patterns in both male and female mice across various diets and stress conditions by assessing RER levels. The RER, denoting the ratio of carbon dioxide (CO_2_) production to oxygen (O_2_) absorption, serves as an indicator of the specific energy source—carbohydrate or fat—that the body metabolizes to fulfill its energy requirements. We observed a notable main effect of sex on the RER in HFD-fed mice (Fig. 3C) (*n* = 4,5; one-way ANOVA; *p* < 0.05). *Post hoc* comparisons revealed marked differences in RERs between male mice and female mice. Specifically, female mice exhibited significantly lower RERs under both the RS and NRS conditions (*n* = 4,5; Tukey’s multiple comparison test; *p* < 0.05). Our observations indicated the absence of any discernible sex or stress effects on RER values in the animals fed a chow diet (Fig. 3D) (*n* = 5; one-way ANOVA; *p* > 0.05). Interestingly, our observations revealed a significant main effect of sex on locomotor activity in mice fed a HFD (Fig. 3E) (*n* = 5; one-way ANOVA; *p* < 0.05). *Post hoc* comparisons revealed that HFD-RS male mice had significantly lower pedestrian locomotion than the HFD-fed female groups (*n* = 4,5; Tukey’s multiple comparison test; *p* < 0.05). We also observed a main effect of sex on pedestrian locomotion among mice fed a normal chow diet (Fig. 3H) (*n* = 5; one-way ANOVA, *p* < 0.05). *Post hoc* analysis revealed that both the RS and NRS groups of female chow-fed mice exhibited greater locomotor activity than the male groups (*n* = 4,5; Tukey’s multiple comparison test, *p* < 0.05). We did not see any effect of stress or sex in either HFD-fed or chow-fed groups in total food consumption (Sup. Figure 2 C and 2D).

### HFD and acute stress have synergistic impacts on behavioral responses, glucose homeostasis, and energy metabolism

**Fig. 4:**
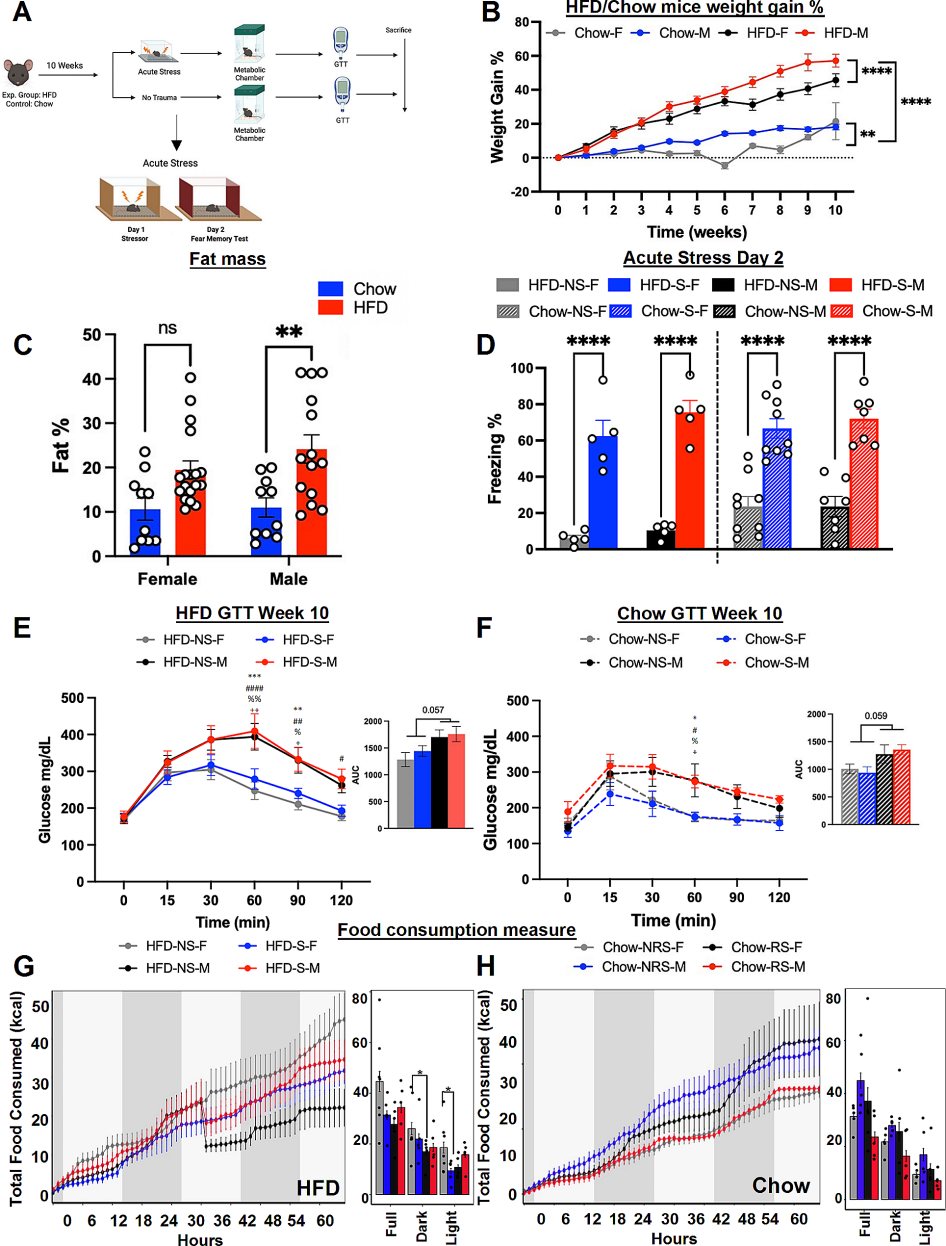
A HFD did not affect acute stress-induced fear behaviors but did cause sex-specific metabolic alterations. **(A)** Experimental design of the comprehensive 10-week study conducted to investigate the role of diet and acute stress. **(B)** Weight gain percentages plotted through 10 weeks of the HFD/chow diet regimen (*n* = 10, two-way ANOVA, F_3,528_ = 188.3, ****p < 0.0001) (*Post hoc* comparison, *n* = 10, Tukey’s multiple comparisons test, *p* < 0.05 from week 2) **(C)** Percent fat mass in HFD/chow-fed mice (*n* = 10; two-way ANOVA, F_1,48_ = 17.69; ***p < 0.001) (*post hoc* comparison, *n* = 10, Tukey’s multiple comparisons test, **p < 0.01). **(D)** Percent freezing in HFD/chow-fed stressed and no stressed groups (*n* = 5, one-way ANOVA, F_7,44_ = 1.388, ****p < 0.0001) (*post hoc* comparison, *n* = 5, Tukey’s multiple comparison test, Chow-NS-F v. Chow-S-F, Chow-NS-M v. Chow-S-M, HFD-NS-F v. HFD-S-F, HFD-NS-M v. HFD-S-M; ****p < 0.0001). **(E)** Plasma glucose levels from the GTT performed at week 10 in HFD-NS/S female and male mice (*n* = 5, two-way ANOVA, F_3,168_ = 18.53, ****p < 0.0001). AUC graph for the week 10 GTT test in HFD/chow-fed female mice (*n* = 5, one-way ANOVA; F_3,16_ = 0.1454, *p* = 0.07). **(F)** Plasma glucose levels from the GTT performed at week 10 in Chow–NS/S female and male mice (*n* = 5, two-way ANOVA, F_3,120_ = 9.057, ****p < 0.0001). AUC graph for the week 10 GTT test in chow-fed mice (*n* = 5, one-way ANOVA, F_3,16_ = 1.206, *p* = 0.41). **(G)** Energy expenditure (EE) (kCal/hr) in HFD-fed S and NS mice (*n* = 5; one-way ANOVA, *p* > 0.05). **(H)** Energy expenditure (EE) (kCal/hr) in chow-fed mice under either the S or NS conditions (*n* = 5; one-way ANOVA, *p* > 0.05)

To investigate the impact of HFD on fear-related behaviors and energy metabolism in response to acute stress, we conducted a comprehensive experimental study following a specific timeline and setup (Fig. 4A). As shown in Fig. 4B, our results showed a significant main effect of sex and diet on the weight gain percentage from baseline (*n* = 10, two-way ANOVA; F_3,528_ =188.3 & F_10,528_ =54.75, *p* < 0.0001). As anticipated, mice fed a HFD exhibited a greater rate of weight gain than control mice fed a chow diet, with discernible differences manifesting as early as the second week of the diet regimen (*n* = 10; Tukey’s multiple comparison test; *p* < 0.0001). However, it is noteworthy that male mice in both diet groups exhibited a notably greater weight gain than their female counterparts within the respective diet groups (*n* = 10, Tukey’s multiple comparison test; *p* < 0.0001). Next, we examined fat mass in these animals at the end of week 10, and two-way ANOVA showed a main effect of diet (Fig. 4C) (*n* = 10; two-way ANOVA; F_1,48_ = 17.69, *p* < 0.05). *Post hoc* analysis further revealed that, exclusively among male mice on HFD, there was a significant increase in the percentage of fat mass compared to that of their counterparts on the Chow diet (*n* = 10, Tukey’s multiple comparison test; *p* < 0.05).

The analysis of freezing behavior in these animals revealed a main effect of stressor (Fig. 4D) (*n* = 5; one-way ANOVA; F_7,44_ = 1.388, *p* < 0.05). *Post hoc* analysis revealed the following differences: Chow-NS-F vs. Chow-S-F, Chow-NS-M vs. Chow-S-M, HFD-NS-F vs. HFD-S-F, HFD-NS-M vs. HFD-S-M (*n* = 5; Tukey’s multiple comparison test; *p* < 0.05). However, we do not see any effect of sex or diet.

GTT tests conducted after the acute stress paradigm revealed a significant main effect of sex and stress in HFD-fed mice (Fig. 4E) (*n* = 5; two-way ANOVA, F_3,168_ =18.53, *p* < 0.05). *Post hoc* comparison further revealed that this sex effect was particularly pronounced during the 60th -120th minute of measurement, specifically between female mice on HFD and male mice on HFD (*n* = 5, Tukey’s multiple comparison test, *p* < 0.05). The area under the curve (AUC) did not illustrate significant difference in female and male mice on HFD, however a trend was observable (*n* = 5; one-way ANOVA, F_3,16_ =3.084, *p* = 0.057). Furthermore, in chow mice, we observed a main effect of sex and stress on blood glucose levels (Fig. 4F) (*n* = 5; two-way ANOVA, F_3,96_ =12.61, *p* < 0.05). *Post hoc* comparison revealed an increase in blood glucose levels in male mice compared to female mice, particularly evident during the 60th -minute interval of measurement (*n* = 5, Tukey’s multiple comparison test, *p* < 0.05). The AUC for the GTT did not show any discernible effect of sex or stress on blood glucose levels, however, we do observe a trend (*n* = 5; one-way ANOVA, F_3,16_ =1.026, *p* = 0.059). Subsequently, we placed the animals in metabolic chambers to quantify changes in metabolic parameters. We observed an effect of sex and stress in EE between male and female mice fed HFD S or NS conditions (Fig. 4G) (*n* = 5; one-way ANOVA, *p* < 0.05). *Post hoc* comparison showed a difference between HFD-S-M and HFD-NS-F groups during dark and light phase. We did not observe a sex effect on EE in mice fed a chow diet (Fig. 4H) (*n* = 5; one-way ANOVA, *p* > 0.05). We further examined additional metabolic parameters, including the RER, total food consumption and locomotor activity, we observed no main effect of sex or stress on the RER or food intake (Sup. Figure 3 A-3D) (*n* = 5; one-way ANOVA, *p* > 0.05). However, we did observe an effect of sex and stress in both HFD and chow groups in locomotor activity (Sup. Figure 3E-3 F) (*n* = 5; one-way ANOVA, *p* < 0.05). *Post hoc comparison* showed group differences between male mice in both stress conditions in comparison to female mice in NS groups.

### Repeated stress impacts myeloid lineage cells within the bone marrow and inflammatory cells in the blood, gWAT, aorta, and heart

To investigate the potential for systemic inflammation resulting from repeated shocks, we initially assessed the expression of proinflammatory genes in the gWAT and liver via quantitative polymerase chain reaction (qPCR). We did not observe any discernible effects of stress or sex on C-C Motif Chemokine Ligand 2 (*CCL2*) gene expression (*n* = 9,10; two-way ANOVA, *p* = 0.15) (Sup. Figure 4 A & 4E). Interestingly, we observed a main effect of sex and stress on the gene expression levels of the proinflammatory cytokine interleukin-1beta (*Il-1β*) (*n* = 9,10; two-way ANOVA, F_1,15_ = 9.796; *p* = 0.0069) and *Il-12p40* (*n* = 9,10; two-way ANOVA, F_1,15_ = 8.352; *p* = 0.011) in HFD-fed mice (Sup. Figure 4B & 4 C). According to our analysis of the liver, sex and stress had no discernible effects on the gene expression levels of *CCL2, Il-1β, Il-12p40* or tumor necrosis factor-alpha (*TNF-α*) (*n* = 9; two-way ANOVA, *p* > 0.05) (Sup. Figure 4E-4 H).

**Fig. 5:**
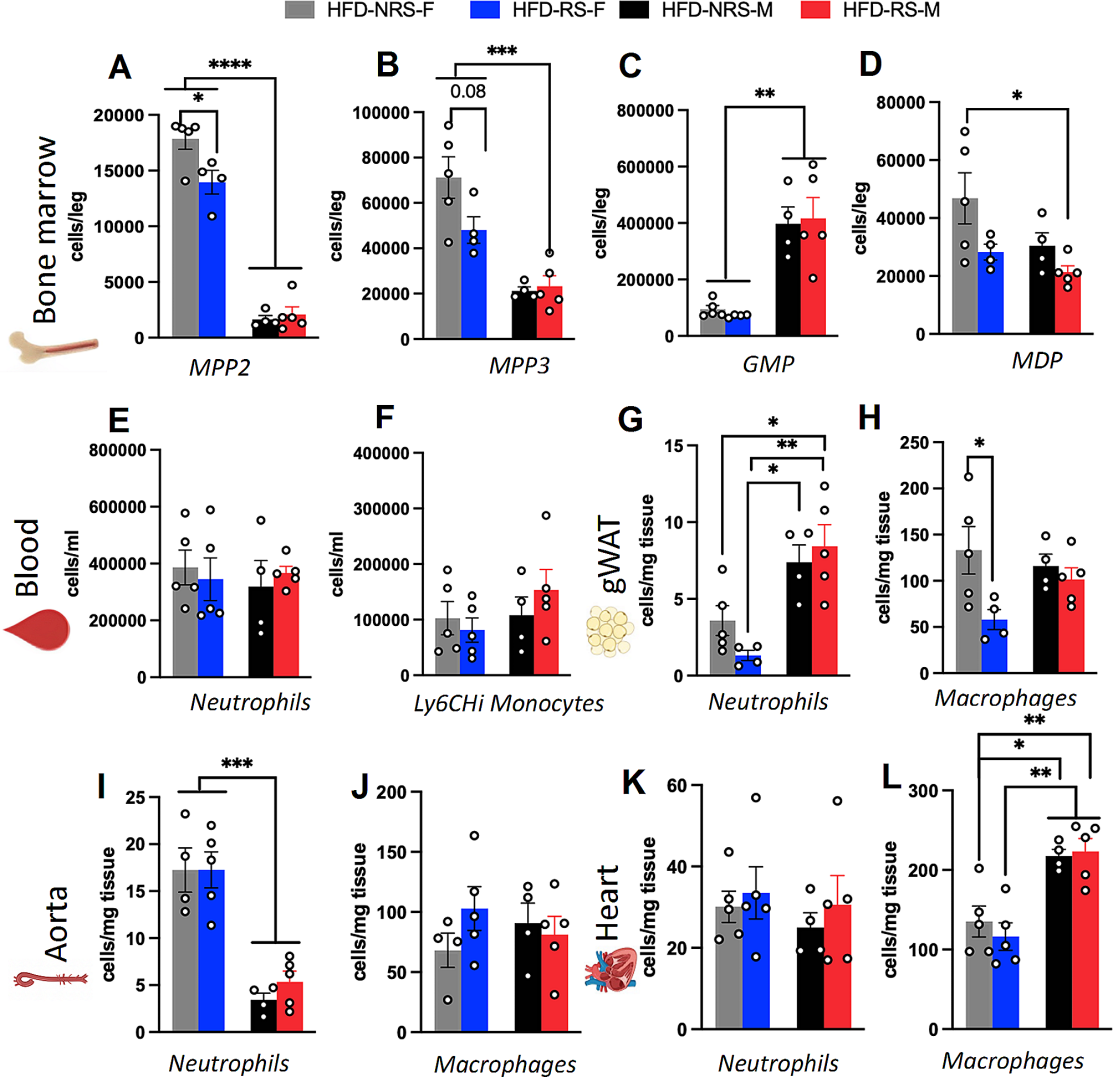
Repeated reminder shocks and a high-fat diet (HFD) induced distinctive alterations in peripheral myeloid lineage cells and inflammatory markers, exhibiting a sexually dimorphic pattern. **(A)** Quantification of the bone marrow population of MPP2 progenitors in HFD-fed mice (*n* = 9; two-way ANOVA, F1,14 = 290.1; ****p < 0.0001) (*post hoc* comparison, *n* = 5; Tukey’s multiple comparison test, *p < 0.05 and ****p < 0.0001). **(B)** Quantification of the MPP3 progenitor population in the bone marrow of HFD-fed mice (*n* = 9; two-way ANOVA; F1,14 = 34.48; ****p < 0.0001) (*post hoc* comparison, *n* = 5; Tukey’s multiple comparison test, *p* = 0.08 and ***p < 0.001). **(C)** Quantification of granulocyte monocyte progenitor (GMP) cells in HFD-fed mice (*n* = 9; two-way ANOVA, F1,14 = 42.52; ****p < 0.0001) (*post hoc* comparison, *N* = 4/5; Tukey’s multiple comparison test, **p < 0.01). **(D)** Quantification of monocyte-dendritic cell progenitors (MDPs) in the bone marrow of HFD-fed mice (*n* = 9; two-way ANOVA, F1,14 = 6.017, *p < 0.05) (*post hoc* comparison, *n* = 5; Tukey’s multiple comparison test, *p < 0.05). **(E)** Quantification of neutrophils in the blood of HFD-fed mice (*n* = 9; two-way ANOVA, *p* > 0.05). **(F)** Quantification of Ly6Chi monocytes in the blood of HFD-fed mice (*n* = 9; two-way ANOVA, *p* > 0.05). **(G)** Quantification of neutrophils in the gonadal white adipose tissue (gWAT) of HFD-fed mice (*n* = 9; two-way ANOVA, F1,14 = 24.65, ***p < 0.001) (*post hoc* comparison, *n* = 5; Tukey’s multiple comparison test, *p < 0.05 and **p < 0.01). **(H)** Quantification of macrophages in the gWAT of HFD-fed mice (*n* = 9; two-way ANOVA; F1,14 = 6.296; *p < 0.05) (*post hoc* comparison; *n* = 4/5; Tukey’s multiple comparison test; *p < 0.05). **(I)** Quantification of aortic neutrophils from HFD-fed mice (*n* = 9; two-way ANOVA; F1,14 = 59.59; ****p < 0.0001) (*post hoc* comparison; *n* = 4/5; Tukey’s multiple comparison test; ***p < 0.001). **(J)** Quantification of macrophages in the aortas of HFD-fed mice (*n* = 4/5; Tukey’s multiple comparison test, *p* > 0.05). **K.** Quantification of neutrophils in the hearts of HFD-fed mice (*n* = 9; two-way ANOVA, *p* > 0.05). **L.** Quantification of macrophages in the hearts of HFD-fed mice (*n* = 9; two-way ANOVA; F1,14 = 32.04; ****p < 0.0001) (*post hoc* comparison; *n* = 4/5; Tukey’s multiple comparison test; *p < 0.05 and **p < 0.01)

Inflammation is an important contributor to metabolic abnormalities and collectively plays a role in the progression of cardiometabolic disorders [68]. Although we did not assess the cardiometabolic disorders phenotype in the present study, the examination of leukocyte abundance in organs pertinent to cardiometabolic disorders provides valuable insights into the potential relevance of our findings to this disease. To address this aspect, we utilized flow cytometric analysis (FACS) to analyze cells collected from the bone marrow, blood, gWAT, aorta and heart tissues of HFD-fed male and female mice that received either RS or NRS, as outlined in the methods section. The gating strategies used for flow cytometry data analysis are shown in Sup. Figure 5A-5E. Multipotent progenitors (MPPs) within the bone marrow, including MPP2 and MPP3, serve as precursors that give rise to both myeloid and lymphoid cell populations. We found a main effect of sex on the MPP2 cell population in the bone marrow of HFD-fed mice (*n* = 9; two-way ANOVA, F_1,14_ = 290.1, *p* < 0.0001) (Fig. 5A). *Post hoc* comparisons revealed a significant reduction in the MPP2 cell population in HFD-RS female mice compared to HFD-NRS male mice (*n* = 5; Tukey’s multiple comparison test, *p* = 0.022) (Fig. 5A). Additionally, we observed a significant decrease in MPP2 cell expression in HFD-fed male mice compared to HFD-fed female mice, irrespective of the presence of stress (*n* = 4/5; Tukey’s multiple comparison test, *p* < 0.0001). We observed a comparable main effect of sex on the population of MPP3s within the bone marrow of HFD-fed mice (*n* = 9; two-way ANOVA, F_1,14_ = 34.48, *p* < 0.0001) (Fig. 5A). Although a *post hoc* analysis revealed a decreasing trend in HFD-RS female mice compared to HFD-NRS female mice, the difference was not statistically significant (*n* = 5, Tukey’s multiple comparison test, *p* = 0.08). There was a significant reduction in MPP3 cell expression in male mice fed a HFD compared to female mice fed the same diet, irrespective of the stress group (*n* = 4/5, Tukey’s multiple comparison test, *p* = 0.003).

Next, upon examination of the downstream products of the MPPs, namely, granulocyte-monocyte precursors (GMPs) and monocyte-dendritic progenitor cells (MDPs), in the bone marrow, we observed a statistically significant main effect of sex (Fig. 5C and D). Hematopoietic stem cells, including MPP2s and MPP3s, progress through stages to generate multipotent common myeloid progenitor cells (CMPs). CMPs further differentiate into GMPs and MDPs, ultimately producing mature leukocytes [69]. Quantification of GMPs in HFD-fed mice revealed a main effect of sex (Fig. 5C) (*n* = 9; two-way ANOVA, F_1,14_ = 42.52, *p* < 0.0001). *Post hoc* comparison revealed a significant decrease in GMPs in HFD-fed female mice compared to their male counterparts (*n* = 5 − 4; Tukey’s multiple comparison test, *p* = 0.001); however, we did not observe any stress effects. Conversely, we observed a main effect of stress on the MDP population in the HFD-fed groups (Fig. 5D) (*n* = 9; two-way ANOVA, F1,14 = 6.017, *p* = 0.057). *Post hoc* comparisons revealed a significant reduction in MDP levels in HFD-RS male mice compared to HFD-NRS female mice (*n* = 5 − 4; Tukey’s multiple comparison test, *p* = 0.0201).

We did not observe any discernible effects of sex or stress on neutrophils or the Ly6Chi monocyte population in the analyzed blood samples (Fig. 5E and F) (*n* = 5 − 4; one-way ANOVA, *p* = 0.72 & *p* = 0.42). Next, we analyzed neutrophil and macrophage populations, which are crucial components of systemic metabolic regulation, in gWAT, the aorta and heart tissue (Fig. 5G and L). We observed a significant main effect of sex and stress on gWAT neutrophil levels (Fig. 5G) (*n* = 9; one-way ANOVA, F_1,14_=24.65; *p* = 0.0002) and a main effect of sex and stress on the macrophage population (Fig. 5H) (*n* = 9; one-way ANOVA, F_1,14_=6.296; *p* = 0.04). *Post hoc* comparisons revealed an increase in neutrophil counts in HFD-fed male mice compared to HFD-RS female mice in the RS and NRS groups (*n* = 5 − 4; Tukey’s multiple comparison test, *p* = 0.01 & *p* = 0.0022) and an increase in macrophage counts in HFD-NRS female mice compared to HFD-RS female mice (*n* = 5; Tukey’s multiple comparison test, *p* = 0.043). In the aorta, we observed a notable main effect of sex and stress on neutrophil counts, indicating the opposite pattern (*n* = 9; two-way ANOVA, F_1,14_ = 59.59, *p* < 0.0001) (Fig. 5I). *Post hoc* analysis revealed significantly greater numbers of neutrophils in HFD-fed female mice than in male mice, irrespective of the stress group (*n* = 5; Tukey’s multiple comparison test, *p* = 0.0009). We did not observe a main effect of sex or stress on macrophage numbers in the aorta (Fig. 5J) (*n* = 5 − 4; one-way ANOVA, F_114_ = 0.00089, *p* = 0.97). We also did not observe any effect of sex or stress on heart neutrophil counts in HFD-fed mice (Fig. 5K) (*n* = 9; two-way ANOVA, F_1,14_=0.491, *p* = 0.49). However, ANOVA revealed a main effect of sex and stress on the number of macrophages in the heart (Fig. 5L) (*n* = 9; two-way ANOVA, F_1,14_=32.04, *p* < 0.0001). *Post hoc* analysis revealed that female mice in the HFD-NRS group had significantly lower levels of macrophages than male mice in the HFD-fed male group did (*n* = 5 − 4; Tukey’s multiple comparison test, *p* = 0.019 & *p* = 0.001). Additionally, female mice in the HFD-RS female group had significantly lower levels of macrophages than male mice in the HFD-fed group (*n* = 5 − 4; Tukey’s multiple comparison test, *p* = 0.007). We extended our analysis to tissues from chow-fed mice, and our observations indicated that sex or stress had minimal impacts on the myeloid cell lineage population and proinflammatory marker levels (Sup. Figure 5 F-5 M).

### Single-nuclei RNA sequencing of the ventromedial hypothalamus revealed sexually dimorphic cellular functions following HFD feeding and repeated stressors

**Fig. 6:**
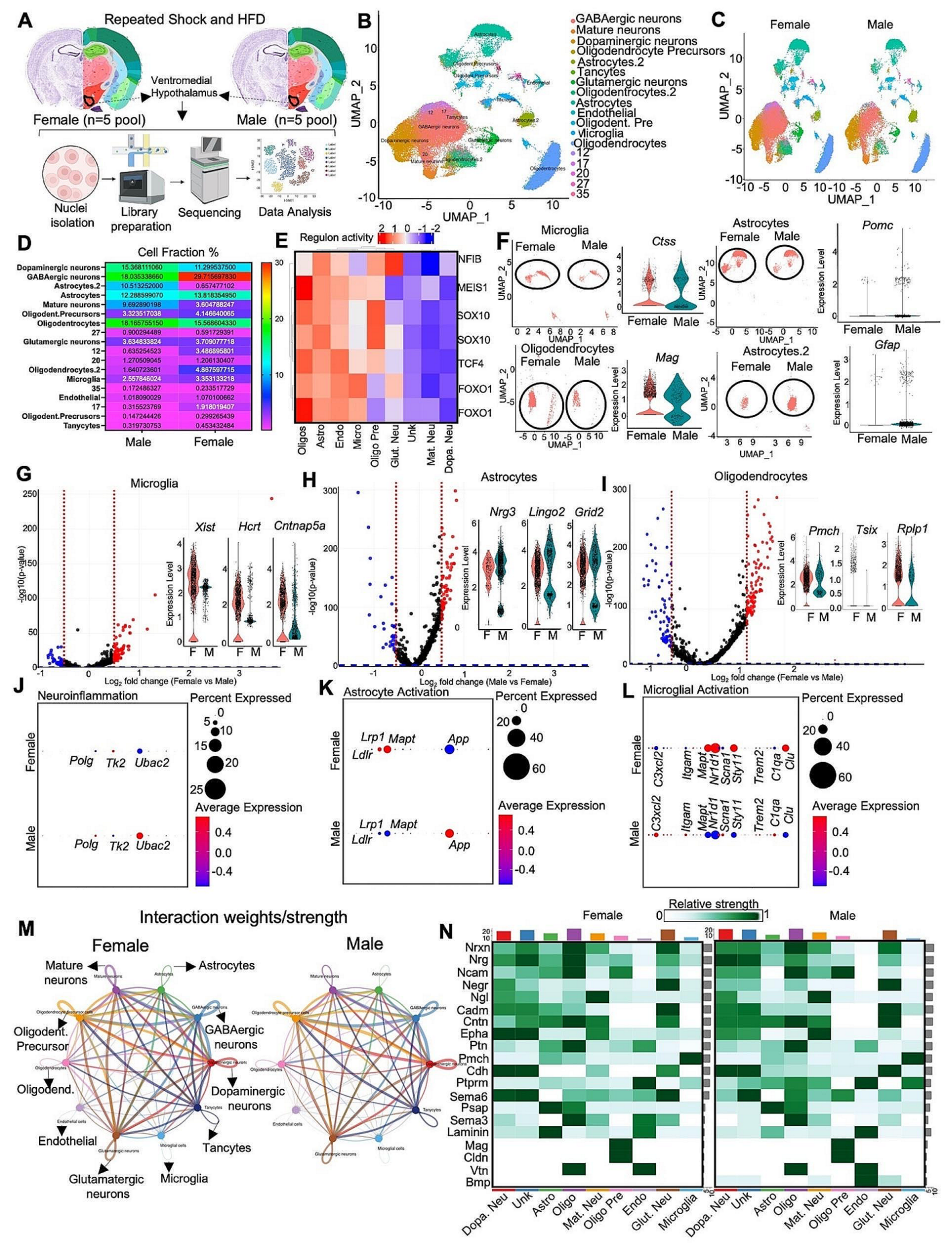
Repeated reminder shock and a high-fat diet (HFD) induced differential changes in the ventromedial hypothalamus (VMH) of male and female mice. **(A)** Illustration outlining the snRNA-seq procedure utilizing the 10X Genomics platform workflow, which involves isolating nuclei from the VMH of male and female mice fed a HFD (*n* = 5 pooled). **(B)** UMAP plot displaying single cells from this study, color coded by cell type, with cell types identified based on the expression of canonical marker genes. **(C)** UMAP plot of single cells from the VMH cohort, color coded by cell type and segregated by sample. **(D)** Heatmap depicting the relative fractions of each cell type in each sample. **(E)** Heatmap illustrating the regulon activity of the indicated transcription factors, indicating the intensity of gene regulation in specific cell types from the VMH of males and females. **(F)** UMAP plot of the indicated cell types from the VMH cohort separated by sample; adjacent violin plots displaying the expression levels of specific genes are shown. **G, H & I.** Volcano plot illustrating differentially expressed genes (DEGs) with fold changes plotted against p values; female vs. male in microglia (G), male vs. female in astrocytes (H), and female vs. male in oligodendrocytes (I). Violin plots highlighting the highly upregulated genes. **J, K & L.** Pathway analysis of DEGs showing neuroinflammation (J), astrocyte activation (K), and microglial activation (L). **M.** Interactome representation based on DEGs from male and female mice indicating the strength of interaction between specific cell types. **N.** Heatmap exhibiting the indicated ligand-cell interactions in male and female mice

As mentioned before, we directed our efforts toward the analysis of VMH cellular functions under conditions of stress and HFD consumption using single-nuclei RNA sequencing (snRNAseq). We performed snRNA-seq to examine the impact of repeated shocks and a HFD on the VMH of both male and female mice (Fig. 6A). Nuclei were isolated from the entire VMH of male and female mice from the HFD-RS group (pooled from 5 mice each). The single-nucleus suspension (*n* ~ 10,000) was subjected to snRNA-Seq using the 10X Genomics platform, and the libraries were sequenced with 1 billion dedicated reads per sample. We utilized the 10X Genomics data processing platform and SeuratV3 to generate cell clusters and identities (refer to Materials and methods) (Fig. 6A). To classify VMH populations based on gene expression, we conducted cluster analysis, as illustrated by uniform manifold approximation and projection (UMAP) plots (Fig. 6B). These plots enabled us to distinguish distinct clusters of GABAergic neurons, mature neurons, dopaminergic neurons, oligodendrocyte precursors, astrocyte populations, glutamatergic neurons, oligodendrocyte populations, endothelial cells, and microglia. Notably, based on the marker genes, we identified two populations, astrocytes, and oligodendrocytes (Fig. 6B). Further examination through UMAP and gene network plots revealed that each cluster uniquely expressed marker genes, demonstrating preferential expression in individual clusters (Sup. Figure S5 A & B). To gain insight into sex differences in VMH remodeling, we generated a cumulative UMAP plot of female and male mice (Fig. 6C). Overall, the UMAP indicated comparable qualitative changes in the relative proportions of VMH clusters between male and female nuclei (Fig. 6C). To characterize the differences in cell fraction between male and female mice, we calculated cluster percentages in relation to the overall combined dataset. We observed variations in astrocytes, oligodendrocytes, microglia, GABAergic neurons, and mature neurons (Fig. 6D). Transcriptional analyses based on gene signatures revealed an active transcriptional network involving transcription factors such as *NFIB, MEIS1, SOX10*, and *FOXO1* in glial cells. These findings suggested that a HFD and repeated stress had significant impacts on the transcriptional program of glial cells (Fig. 6E). Subsequent analysis included subsetting of glial cells, including microglia, astrocytes, and oligodendrocytes. This analysis revealed increased percentages of microglia and oligodendrocytes in female VMH samples, marked by *Ctss* and *Mags*, and an increased astrocyte population in male VMH samples. Interestingly, the neuropeptide *Pomc* and the astrocyte marker *Gfap* were notably more abundant in male astrocytes than in female astrocytes (Fig. 6F). To delineate transcriptional differences in glial cell populations between male and female mice, we performed differential gene expression analysis on oligodendrocytes, microglia, and astrocytes. The volcano plot in Fig. 6G illustrates the highly upregulated genes, including *Xist, Hcrt*, and *Ctnap5a*, in the microglia of female VMHs compared to those in male VMHs. Among the astrocytic population, the highly significant genes in the male VMH were *Nrg3, Lingo2*, and *Grid2* (Fig. 6H). In the pooled female VMH cohort, the oligodendrocyte population consisted of genes such as *Pmch, Tsix*, and *Rplp1* (Fig. 6I). To correlate the gene expression profiles with pathway analysis, we performed Gene Ontology (GO) analysis of the DEGs in the overall neuronal population, astrocytes, and microglia; the results were correlated with cell type activation pathways. Overall neuroinflammation was comparable between male and female VMH samples (Fig. 6J). However, male pooled VMH samples presented an increase in *App* and *Ldlr* expression, while female VMH samples presented an increase in *Lrp1* and *Mapt* expression, indicative of differential activation of astrocyte lipid metabolism (Fig. 6K). Consistent with previous results, female VMH samples showed overall activation of microglia, with increased expression of *Nr1d1, Sty11*, and *Clu* (Fig. 6L). In line with these observations, cell cluster expression associated with genome-wide association study (GWAS) traits was examined using the MAGMA program, which revealed that female mice were more strongly correlated with Alzheimer’s disease frequency than male mice under conditions of repeated stress and a HFD (Sup. Figure 5 C).

To further study how neuronal and non-neuronal cells interact in male and female mice under repeated stress and HFD conditions, we performed cell‒cell communication (ligand‒receptor interaction) analysis using CellChat [70]. We observed markedly more active communication between cell types in female VMH samples than in male VMH samples (Fig. 6M and Sup. Figure 5D). The female VMH samples also exhibited strong autocrine effects on mature neurons, with robust interactions between mature neurons, glutamatergic neurons, and tanycytes (Fig. 6M). Oligodendrocyte precursor connections were comparable between male and female VMH samples. In female VMH samples, oligodendrocytes exhibited stronger connections with glutamatergic neurons, tanycytes, dopaminergic neurons, and astrocytes (Fig. 6M). VMH samples from female mice fed a HFD and subjected to repeated stress also exhibited stronger connections from microglia to glutamatergic neurons, oligodendrocyte precursors, and astrocytes (Fig. 6M). The connections from tanycytes were similar between male and female VMH samples, while dopaminergic neuronal connections with oligodendrocyte precursors were stronger in male VMH samples (Fig. 6M). We further analyzed highly expressed ligands from our dataset, examining their interactions with various cell types, and visualized the results in a heatmap (Fig. 6N). A heatmap was constructed to show sex-specific differences in cellular responses to ligands, with factors such as *Negr* and *Epha* showing stronger interactions with dopaminergic neurons, *Sema6* with dopaminergic and glutamatergic neurons, and laminin with astrocytes in female VMH samples (Fig. 6N). In the male VMH cohort, the intensity of ligand and cellular connections was overall more pronounced, with *Cntn* indicating dopaminergic neurons, *Epha* indicating mature neurons, *Cdh* indicating glutamatergic neurons, *Ptprm* indicating astrocytes, oligodendrocytes, mature neurons, and microglia; *Sema6* indicating oligodendrocyte precursors; and *Bmp* indicating endothelial cells (Fig. 6N).

In conclusion, our snRNA-seq data represent the first comprehensive insight into the impact of repeated stress and a HFD on the VMH in male and female mice. Although the observed sex differences were not overtly pronounced, substantial alterations in the transcriptomic state of glial cells were identified between male and female samples. These changes in the VMH have the potential to regulate neuronal function, thereby influencing behavior and energy metabolism.

## Discussion

Our research revealed a previously unexplored impact of diet and repeated stress on fear-related behaviors, accompanied by various metabolic, immune, and hypothalamic changes. Male mice fed a HFD exhibit decelerated fear extinction, while female mice continue to extinguish fear. HFD-RS female mice exhibited heightened glucose intolerance levels by week 14 of diet and stress exposure. Female mice fed a HFD had a lower respiratory exchange ratio, indicating a reduced reliance on carbohydrates. Male mice fed a HFD exhibit greater energy expenditure but lower locomotor activity. Analysis of peripheral genes revealed sex-specific changes in multipotent progenitor cells and immune cells in various tissues. Single-nucleus RNA sequencing of the ventromedial hypothalamus revealed cell type-specific differences, highlighting diverse pathways involved in fear-related responses and metabolic and immune dysfunction.

Previous research has indicated a link between diet, particularly HFD, and cognitive functions, extending it to fear memory extinction [21]. We observed that male mice on the HFD displayed impaired fear memory extinction, as evidenced by their failure to exhibit a significant reduction in freezing behavior compared to that of the mice in the repeated shock group at week 14. This finding suggested that a HFD inhibits fear extinction processes in male mice. These findings are consistent with studies that have proposed that impaired extinction of fear memories associated with trauma is crucial for the development of PTSD symptoms and is directly correlated with symptom severity [71–74]. Diet could be a risk factor for impaired extinction learning in PTSD patients, and further investigations of this link is needed.

Further, our exploration of the effects of diet and stress on weight gain and blood glucose levels revealed sex-specific differences. While both male and female mice on the HFD exhibited increased weight gain over the 14-week period, males displayed a greater rate of weight gain than females, regardless of the stress condition. In chow-fed animals, however, we observed not just sex difference but a stress effect in females, with repeated stressed females showing a higher rate of weight gain than females in the no repeated stress group. This could allude to development of stress induced weight gain in female mice, revealing clinical aspects of PTSD and eating disorder comorbidity [75, 76].

Additionally, glucose homeostasis were influenced by both stress and sex. Unsurprisingly, we observed that HFD-fed male mice had higher blood glucose levels when they were injected with a bolus of glucose than their female counterparts. Remarkably, with continued exposure to stressors, blood glucose levels increased in HFD-fed females to levels observed in HFD male mice by week 14. However, chow fed animals displayed sex differences in blood glucose levels but not a stress effect by week 10. This sex difference also ceases to be observed by week 14. As mounting evidence indicates associations between PTSD and diabetes [77, 78], our results suggest complex interactions between diet, sex, and stress in modulating metabolic outcomes. These disruptions lead to increased visceral adiposity, inflammation, and insulin resistance, which might, in turn, perpetuate further irregularities in HPA axis functioning, creating a harmful cycle of deteriorating health [79].

In a state of homeostasis, food consumption generally aligns with energy expenditure. However, when rodents are exposed to a HFD or experience repeated stress, the central and peripheral mechanisms that regulate energy balance can be altered, leading to hyperphagia and greater accumulation of adipose tissue [80, 81]. In our studies, we investigated the effects of metabolic parameters by employing indirect calorimetry measurements. Males on a HFD exhibit greater energy expenditure, contrary to the findings of previous studies by Huang et al. [82], which showed greater energy expenditure in HFD-fed female mice than in male mice. This effect could be attributed to the effects of both the short-term stress induced by SEFL in NRS male mice fed a HFD and RS male mice fed HFD. This is also an extremely interesting finding given that we observed a decrease in locomotion in these HFD-fed male RS mice. More robust studies are required to understand the increased energy expenditure observed without the physical demand by locomotor activity. Studies have also shown that the RER decreases with HFD [83], but we see that this effect occurs only in female mice fed a HFD, emphasizing the need to rethink the importance of studying sex differences.

The findings from acute stress-induced experiments indicate that HFD-induced weight gain is associated with both sex and diet effects. Moreover, fat mass was significantly greater in males fed a HFD than in males fed chow, suggesting that the impact of a HFD on weight gain and fat mass might be more pronounced in males. However, we did not observe any diet effects on freezing behavior. Additionally, we observed no effect of stress but only sex on glucose sensitivity. Since the diet regimen was administered for a shorter duration and the stressor would be considered very mild in the acute stress paradigm, this could explain why we did not observe any effects of stress in freezing or GTT.

Neutrophils in the gWAT of male mice fed a HFD may actively contribute to obesity-related inflammation [84]. Neutrophils play a key role in the development of vascular diseases such as aortic aneurysms [85, 86] and inflammation-induced atherosclerosis [87, 88]. Increased levels of neutrophils in the aortas of HFD-fed female mice could indicate the manifestation of cardiovascular pathologies. However, increased numbers of macrophages in the heart tissue of HFD-fed male mice could indicate the initiation of heart inflammation [89]. While our studies have implicated a sex-specific manifestation of comorbid pathologies in HFD-fed mice, delineation of the effect of stress is lacking. However, further studies are needed to determine the working mechanisms involved in the development of these various cardiometabolic diseases.

Hematopoietic stem cells (HSCs) in the bone marrow are rare and mostly quiescent and divide infrequently in a healthy state [90, 91]. Hematopoiesis is known to be impacted by a HFD [92, 93] and various forms of stressors [94, 95]. The majority of continuous blood cell production is carried out by downstream multipotent progenitors (MPPs) [96, 97]; MPP2s exhibit a propensity to differentiate into lymphocytes and megakaryocytes, whereas MPP3s exhibit a propensity to differentiate into monocytes and granulocytes [69]. MPPs sustain hematopoiesis by giving rise to common myeloid progenitors (CMPs), granulocyte–monocyte progenitors (GMPs) and myeloid dendritic progenitors (MDPs) [69, 98]. We observed an effect of stress on the MPPs in only HFD-fed female mice, while in male mice, diet alone caused a reduction in the MMP levels. These findings fit well with the observed increase in the next hierarchical product of hematopoiesis, GMPs, in HFD-fed male mice and the increase in MDPs in HFD-NRS female mice. Further studies are essential to tease apart the changes in the hematopoietic continuum in response to stress challenges.

The snRNA-seq analysis of the VMH from male and female mice exposed to a HFD with repeated stress (RS-HFD) revealed the impact of both repeated stress and HFD on various cell types within the VMH, potentially influencing metabolic phenotypes. Data obtained from pooled VMH regions of male and female RS mice indicated differences in cell fractions among both neuronal and nonneuronal populations. The analysis based on regulon activity and transcriptional signatures of VMH cell types revealed an active transcriptional state in glial cells, including oligodendrocytes, astrocytes, microglia, and oligodendrocyte precursors. Male mice exhibited a greater astrocytic state with increased expression of *Gfap* and *Pomc*, while female mice showed increased expression of the genes encoding orexin (*Hcrt)* in microglia and the gene encoding the pro-melanin concentrating hormone (*Pmch*) in oligodendrocytes. Orexin and *Pmch* are neuropeptides that play major roles in wakefulness [99–101], and in addition to their actions in the central nervous system (CNS) [102, 103], they also regulate appetite, feeding, gastrointestinal mobility, energy balance, metabolism, blood pressure, neuroendocrine, and reproductive functions in various peripheral organs [100, 104–106]. GWAS analysis revealed greater expression of Alzheimer’s disease in female mice than in male mice. Microglia are implicated in diabetic and Alzheimer’s disease phenotypes [107–110], suggesting that RS-HFD may contribute to metabolic disorders in females. Similarly, our cell‒cell communication analysis showed that female mice also exhibited stronger connections from microglia to glutamatergic neurons, oligodendrocyte precursors, and astrocytes.

Our findings are in line with studies that have shown that stress and stress-associated disorders such as PTSD can cause disruptions in the neuroendocrine [111, 112], sympathetic [113, 114], metabolic [115–117], and inflammatory systems [118–120] and lead to adverse changes in lifestyle that further contribute to deteriorating health [121–124]. Stress disrupts the established equilibrium, triggering physiological and metabolic adaptations in the body to address the demands that hinder homeostasis. Activating the HPA axis [125] and the sympathetic nervous system (SNS) [126] facilitates these adaptations. Subsequent exposure to repeated stressors following an initial extreme event can impose an excessive burden on these physiological systems, potentially resulting in various disease states as individuals struggle to cope with sustained stress. The cascade of events stemming from this accumulated allostatic load contributes to the emergence of metabolic and inflammatory disorders [127]. Negative alterations in health can also be linked to unhealthy lifestyle choices, such as a high-fat diet, which may stem from a fear of encountering environmental triggers that elicit symptoms of PTSD. This dietary preference might serve as a coping mechanism adopted by the individual [128]. Our finding that a HFD and repeated stressors alter the number of immune cells is in line with alterations in the immune-modulating stress hormone cortisol [129], which in turn affects cognitive function [20–22, 130, 131] and promotes inflammation [132, 133]. Overall, our study provides the groundwork for further comprehension of the mechanisms underlying metabolic health and the onset of stress-related disorders. Subsequent research is essential to explore the underlying mechanisms driving these sex-specific effects, encompassing neural, hormonal, and molecular factors, providing a clearer understanding of these complex interactions.

### Perspective and significance

The findings revealed in this research study demonstrate sexual dimorphism in fear extinction, a pivotal aspect of the development of PTSD. Recognizing the sex-specific nuances in fear-related responses is essential for individualizing interventions and therapeutic strategies. Furthermore, the exploration of potential connections between diet, sex, and stress in influencing metabolic outcomes, such as glucose sensitivity, energy expenditure, the RER, and feeding, suggests multifaceted interactions with broader implications for overall health and well-being. This finding implies that sex-specific pathways play a significant role in the development of fear-related disorders and metabolic dysfunction. However, these dysfunctions do not occur in isolation; rather, they involve additional pathways that contribute to the complexity of disease development. One notable pathway is immune regulation, which our study revealed to be disrupted in various peripheral tissues, such as bone marrow, blood, gWAT, aorta and heart tissues. snRNAseq of the VMH also revealed sex-specific neuroinflammatory mechanisms triggered by HFD feeding and repeated stress. Acknowledging these sex-specific nuances is critical for developing personalized interventions that account for individual variations in responses to diet and stress, paving the way for more effective and tailored therapeutic approaches.

## Conclusion

Our ongoing research emphasizes how combining a high-fat diet and stress influences behavioral and metabolic functions differently depending on sex. Our results provide insight into the importance of investigating male and female differences across various levels of study.

### Electronic supplementary material

Below is the link to the electronic supplementary material.

Supplementary Material 1

## Data Availability

This study did not generate new unique reagents. All the data reported in this paper will be shared by the lead contact upon request This paper does not report any original code. Any additional information required to reanalyze the data reported in this work paper is available from the lead contact upon request

## Declarations

### Lead contact

Further information and requests for resources and reagents should be directed to and will be fulfilled by the Lead Contact, Dr. Abha K. Rajbhandari (abha.rajbhandari@mssm.edu).
