# Supplementary material for: Sex-specific role of high-fat diet and stress on behavior, energy metabolism, and the ventromedial hypothalamus
Source: Biol Sex Differ. 2024 Jul 15;15:55. doi: 10.1186/s13293-024-00628-w (PMC11247790; doi:10.1186/s13293-024-00628-w)
Supplement: Supplementary file 1 — Supplementary Material 1 [file 13293_2024_628_MOESM1_ESM.docx]

**Supplemental Information**

**Key Resources Table**

| **REAGENT or RESOURCE** | **SOURCE** | **IDENTIFIER** |
| --- | --- | --- |
| **Diet** | | |
| Chow | Laboratory Rodent Diet | Cat# 3005659-220 |
| High Fat Diet | Research Diets Inc. | Cat#D12492 |
| **Antibodies** | | |
| Biotin anti-mouse CD45 Antibody | BioLegend | Cat# 103103 |
| Biotin anti-mouse CD11b | BioLegend | Cat# 133307 |
| Biotin anti-mouse CD90.2 (Thy1.2) Antibody | BioLegend | Cat# 105304 |
| Biotin anti-mouse CD45R/B220 | BioLegend | Cat# 133307 |
| Biotin anti-mouse CD19 Antibody | BioLegend | Cat# 115503 |
| Biotin anti-mouse CX3CR1 Antibody | BioLegend | Cat# 149018 |
| Biotin anti-mouse Ly-6G Antibody | BioLegend | Cat# 47003 |
| Biotin anti-mouse Ly-6C Antibody | BioLegend | Cat# 47003 |
| Biotin anti-mouse F4/80 Antibody | BioLegend | Cat# 12305 |
| Biotin anti-mouse I-A/I-E Antibody | BioLegend | Cat# 107603 |
| Biotin anti-mouse CD64 (FcγRI) Antibody | BioLegend | Cat# 139318 |
| Biotin anti-mouse CD49b Antibody | BioLegend | Cat# 108903 |
| Biotin anti-mouse TER-119 Antibody | BioLegend | Cat# 116203 |
| Biotin anti-mouse CD11c Antibody | BioLegend | Cat# 117303 |
| Purified anti-mouse CD127 Antibody | BioLegend | Cat# 158203 |
| Purified anti-mouse CD117 Antibody | BioLegend | Cat# 105801 |
| Biotin anti-mouse Ly-6A/E Antibody | BioLegend | Cat# 122504 |
| Biotin anti-mouse CD135 Antibody | BioLegend | Cat# 135307 |
| Biotin anti-mouse CD48 Antibody | BioLegend | Cat# 103409 |
| Biotin anti-mouse CD150 Antibody | BioLegend | Cat# 115907 |
| Anti-mouse CD34 Monoclonal Antibody | eBioscience (Thermo Fischer) | Cat# 14034182 |
| Biotin anti-mouse CD16/32 Antibody | BioLegend | Cat# 101303 |
| Biotin anti-mouse CD115 Antibody | BioLegend | Cat# 135507 |
| Anti-mouse BrdU Monoclonal Antibody | eBioscience  (Thermo Fisher) | Cat# 11507142 |
| Anti-mouse feeder cell antibody | Miltenyi Biotec | Cat# 130120166 |
| Biotin anti-mouse Podoplanin Antibody | BioLegend | Cat# 127403 |
| Biotin anti-mouse CD31 Antibody | BioLegend | Cat# 102404 |
| **Chemicals, peptides, and recombinant proteins** | | |
| Acetic Acid |  |  |
| Insulin | Thermo Fischer | Cat# 12585014 |
| Glucose | Sigma | Cat# G8769-100ML |
| Phenol‒chloroform |  |  |
| Diethylpyrocarbonate (DEPC) water |  |  |
| RBC lysis buffer | BioLegend | Cat# 420301 |
| Collagenase from Clostridium histolyticum,  Type I | Sigma Aldrich | Cat# C0130-1G |
| Collagenase from Clostridium histolyticum  XI | Sigma Aldrich | Cat# C7657-100MG |
| Deoxyribonuclease I from bovine pancreas | Sigma Aldrich | Cat# D4513-1VL |
| Hyaluronidase from bovine testes | Sigma Aldrich | Cat# H3506-100MG |
| Live/Dead Blue | Thermo Fischer | Cat# L23105 |
| Albumin bovine, fraction V, ≥98% | MP BIOMEDICALS | SKU: 0216006991 |
| UltraPure™ 0.5 M EDTA, pH 8.0 | Thermo Fisher | Cat# 15575020 |
| **Critical commercial assays** | | |
| cDNA reverse transcription kit | Applied Biosystems | Cat# 4368814 |
| PCR SYBR green master mix | Diagenode |  |
| Next GEM Single cell 3’GEX Reagent kit | 10x Genomics | Cat# 1000092 |
| **Experimental models: Organisms/strains** | | |
| C57BL/6J | The Jackson Laboratory | Stock #: 000664 |
| **Software and algorithms** | | |
| EthoVision XT software | Noldus | https://www.noldus.com/ethovision-xt |
| CalR software | Mina et al. | RRID: SCR_015849  https://CalRapp.org |
| FlowJo software | BD Biosciences | RRID:SCR_008520 |
| Cell Ranger v3.0.2 software | 10x Genomics | N/A |
| pySCENIC | github.com/aertslab/pySCENIC | doi:10.1038/nmeth.4463 |
| GRNBoost2 | Arboreto framework | doi: 10.1093/bio  informatics/bty916 |
| GraphPad Prism | GraphPad Software | RRID:SCR_002798 |
| **Other** | | |
| High Fat Diet (60%kCal fat) | Research Diets | N/A |
| Chow Diet (3%kCal fat) | Research Diets | N/A |
| Fear Conditioning Chambers | Med Associates | N/A |
| Open field light gradient box | Nagai et al. | N/A |
| Glucometer | AlphaTrack 2 | N/A |
| Metabolic Chamber-Comprehensive Lab Animal Monitoring System (CLAMS) | Columbus Instruments | N/A |
| Applied Biosystem (ABI) qPCR machine | Thermo Fischer | QuantStudio 6 Flex System |
| 100 µm cell strainer |  |  |
| FACS Machine |  |  |
| Singulator instrument | S2 Genomics | https://s2genomics.com/singulator-100/ |
| 10x Chromium platform | 10x Genomics | https://www.10xgenomics.com/platforms/chromium |

**Primer Targets**

| gWAT | | |
| --- | --- | --- |
| Target | Forward Primer Sequence | Reverse Primer Sequence |
| Collagen type I alpha 1 chain (COL1A1) | GCTCCTCTTAGGGGCCACT | CCACGTCTCACCATTGGGG |
| Collagen type III alpha 1 chain (COL3a1) | CTGTAACATGGAAACTGGGGAAA | CCATAGCTGAACTGAAAACCACC |
| Collagen type IV alpha 1 chain (COL4A1) | TCCGGGAGAGATTGGTTTCC | CTGGCCTATAAGCCCTGGT |
| Collagen type V alpha 1 chain (COL5A1) | CTCCAACACCTCCAATCCAG | GTCCTCCAATCCCCTCAAAG |
| Collagen type VI alpha 1 chain (COL6A1) | CTGCTGCTACAAGCCTGCT | CCCCATAAGGTTTCAGCCTCA |
| Collagen type XIV alpha 1 chain (COL14A1) | TTTGGCGGCTGCTTGTTTC | CGCTTTTGTTGCAGTGTTCTG |
| Collagen type XV alpha 1 chain (COL15A1) | CCCATTACCCTCGTCTGTGTC | CTGAAGAAGGTCGGTGGGATG |
| Collagen type XVI alpha 1 chain (COL16A1) | GAGAGCGAGGATACACTGGC | CTGGCCTTGAAATCCCTGG |
| Interleukin 6 (IL-6) | GCTACCAAACTGGATATAATCAGGA | CCAGGTAGCTATGGTACTCCAGAA |
| Monocyte chemoattractant protein 1 (MCP-1) | CATCCACGTGTTGGCTCA | GATCATCTTGCTGGTGAATGA |
| Tumor necrosis factor alpha (TNF-) | TCTTCTCATTCCTGCTTGTGG | GGTCTGGGCCATAGAACTGA |
| Interleukin 1 beta (IL-1) | AGAAGCTGTGGCAGCTACCTG | GGAAAAGAAGGTGCTCATGTCC |
| Interleukin 10 receptor subunit alpha (IL-10R) | GCGTGACTCTGAAAGCAATG | TGAGAACTTCCGGATGGAAA |
| Interleukin 12 subunit p40 (IL-12p40) | ACCTGCCCAACTGCCGAGGA | CTGCCGTGCTTCCAACGCCA |
| Interleukin 12 subunit p35 (IL-12p35) | CTTAGCCAGTCCCGAAACCT | TTGCTGCCGTGTGATGTCT |
| Interferon gamma (IFN-) | TTGCCAAGTTTGAGGTCAACAAC | CGAATCAGCAGCGACTTCTT |
| Interleukin 17F (IL-17F) | CAGGGAGAGCTTCATCTGTGT | GCTGAGCTTTGAGGGATGAT |
| Interleukin 17A (IL-17A | CCCAGGAAGACATACTCAGAAGAAAA | GCAAGTCCCAACATCAACAG |
| Interleukin 23 subunit p19 (IL-23p19) | TCCCTACTAGGACTCAGCCAAC | TGGGCATCTGTTGGGTGT |
| Glucose transporter 1 (GLUT1) | \| TCAACACGGCCTTCACTG \| \| --- \| \|  \| \|  \| | \| CACGATGCTCAGATAGGACATC \| \| --- \| \|  \| \|  \| |
| Glucose transporter 2 (GLUT2) | \| TGTGCTGCTGGATAAATTCGCCTG \| \| --- \| \|  \| \|  \| | \| AACCATGAACCAAGGGATTGGACC \| \| --- \| \|  \| \|  \| |
| Glucose transporter 3 (GLUT3) | \| TTCTGGTCGGAATGCTCTTC \| \| --- \| \|  \| \|  \| | \| AATGTCCTCGAAAGTCCTGC \| \| --- \| \|  \| \|  \| |
| Glucose transporter 4 (GLUT4) | \| GTAACTTCATTGTCGGCATGG \| \| --- \| \|  \| \|  \| | \| AGCTGAGATCTGGTCAAACG \| \| --- \| \|  \| \|  \| |
| Activating transcription factor 4 (ATF4) | GATGGGTTCTCCAGCGACAAG | CCGGAAAAGGCATCCTCCTTC |
| C/EBP homologous protein (CHOP) | GCTGTGGTAGTGAGCTGTTGCA | CACAGCCCAGGTATGGAATCA |
| X-box binding protein 1 (XBP1) | GAACACGCTTGGGAATGGACAC | AGAAAGGGAGGCTGGTAAGGAAC |
| G protein-coupled receptor 78 (GPR78) | GTCTGCTTCGTGTCTCCTCCTG | TCCTCCTTCTTGTCCTCCTCCTC |

| Liver | | |
| --- | --- | --- |
| Target | Forward Primer Sequence | Reverse Primer Sequence |
| Interleukin 6 (IL-6) | GCTACCAAACTGGATATAATCAGGA | CCAGGTAGCTATGGTACTCCAGAA |
| Monocyte chemoattractant protein 1 (MCP-1) | CATCCACGTGTTGGCTCA | GATCATCTTGCTGGTGAATGA |
| Tumor necrosis factor alpha (TNF-) | TCTTCTCATTCCTGCTTGTGG | GGTCTGGGCCATAGAACTGA |
| Interleukin 1 beta (IL-1) | AGAAGCTGTGGCAGCTACCTG | GGAAAAGAAGGTGCTCATGTCC |
| Interleukin 10 receptor subunit alpha (IL-10R) | GCGTGACTCTGAAAGCAATG | TGAGAACTTCCGGATGGAAA |
| Interleukin 12 subunit p40 (IL-12p40) | ACCTGCCCAACTGCCGAGGA | CTGCCGTGCTTCCAACGCCA |
| Interleukin 12 subunit p35 (IL-12p35) | CTTAGCCAGTCCCGAAACCT | TTGCTGCCGTGTGATGTCT |
| Interferon gamma (IFN-) | TTGCCAAGTTTGAGGTCAACAAC | CGAATCAGCAGCGACTTCTT |
| Interleukin 17F (IL-17F) | CAGGGAGAGCTTCATCTGTGT | GCTGAGCTTTGAGGGATGAT |
| Interleukin 17A (IL-17A | CCCAGGAAGACATACTCAGAAGAAAA | GCAAGTCCCAACATCAACAG |
| Interleukin 23 subunit p19 (IL-23p19) | TCCCTACTAGGACTCAGCCAAC | TGGGCATCTGTTGGGTGT |
| Glucose transporter 1 (GLUT1) | TCAACACGGCCTTCACTG | CACGATGCTCAGATAGGACATC |
| Glucose transporter 2 (GLUT2) | TGTGCTGCTGGATAAATTCGCCTG | AACCATGAACCAAGGGATTGGACC |
| Glucose transporter 3 (GLUT3) | TTCTGGTCGGAATGCTCTTC | AATGTCCTCGAAAGTCCTGC |
| Glucose transporter 4 (GLUT4) | GTAACTTCATTGTCGGCATGG | AGCTGAGATCTGGTCAAACG |
| Activating transcription factor 4 (ATF4) | GATGGGTTCTCCAGCGACAAG | CCGGAAAAGGCATCCTCCTTC |
| C/EBP homologous protein (CHOP) | GCTGTGGTAGTGAGCTGTTGCA | CACAGCCCAGGTATGGAATCA |
| X-box binding protein 1 (XBP1) | GAACACGCTTGGGAATGGACAC | AGAAAGGGAGGCTGGTAAGGAAC |
| G protein-coupled receptor 78 (GPR78) | GTCTGCTTCGTGTCTCCTCCTG | TCCTCCTTCTTGTCCTCCTCCTC |
| Mfibronectin | AAACTCGACGCTCCCACTAACCTC | CTTCTCCTGCCGCAACTACTGTGA |
| Transforming growth factor beta 1 (TGF1) | CTCCCGTGGCTTCTAGTGC | GCCTTAGTTTGGACAGGATCTG |
| Fibronectin 1 (FN1) | GATGTCCGAACAGCTATTTACCA | CCTTGCGACTTCAGCCACT |
| Stearoyl-coenzyme A desaturase 1 (SCD1) | TTCCCTCCTGCAAGCTCTAC | CAGAGCGCTGGTCATGTAGT |
| Fatty acid synthase (FASN) | TGCTCCCAGCTGCAGGC | GCCCGGTAGCTCTGGGTGTA |
| Sterol regulatory element binding protein 1C (SREBP1C) | GGAGCCATGGATTGCACATT | GGCCCGGGAAGTCACTGT |

**Supplementary Figures**

**
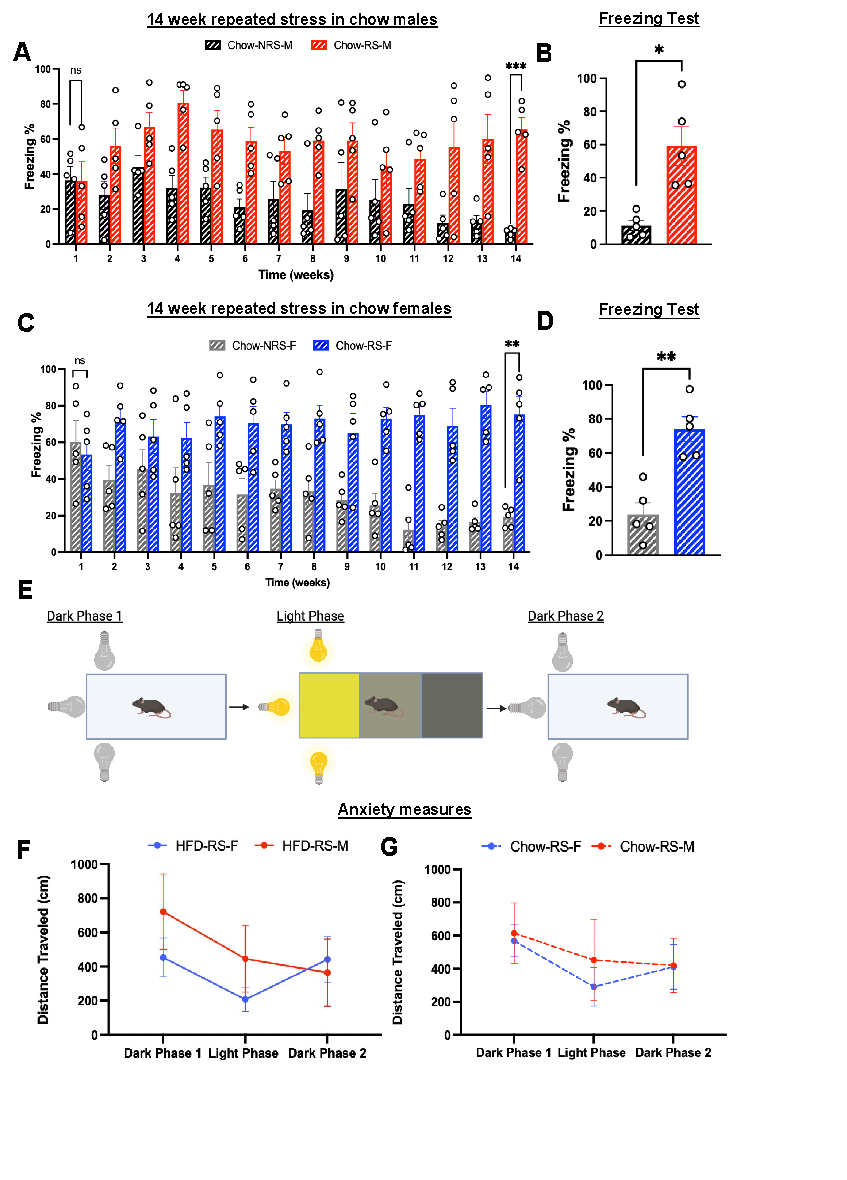
**

**Supplemental Fig 1. Chow fed mice extinguish fear memory and while neither diet nor repeated shocks have an effect on anxiety-like phenotypes. A.**  Percent freezing of chow–fed male mice during 14 weeks of RS/NRS treatment (n=5; two-way ANOVA, F1,112 =89.76; ****p<0.0001) (*post hoc* comparison at week 14, N=5; Sidak’s multiple comparison test, ***p<0.001). **B.** Freezing percentage in chow-fed male mice during freezing test conducted after 14 weeks (n=5, two-tailed paired t test; t=3.586, *p<0.05). **C.** Percent freezing of chow–fed female mice after 14 weeks of RS/NRS treatment (n=5; two-way ANOVA, F1,112 =161.1; ****p<0.0001) (*post hoc* comparison, n=5; Sidak’s multiple comparison test, **p<0.01). **D.** Freezing percentage in chow-fed female mice during freezing test conducted after 14 weeks (n=5, two-tailed paired t test; t=4.771, **p<0.01). **E.** Experimental design of an open field light-gradient task. **F.** Average distance travelled during the three phases of the behavioral task in HFD fed RS female and male mice (N=5, Two way ANOVA, F_1,24_ = 1.1, p=0.29). **G.** Average distance travelled during the three phases of the behavioral task in chow-fed RS male and female mice (N=5, Two way ANOVA, F_1,24_ = 0.33, p=0.57).

**Sup. Fig. 2. HFD has no effect on insulin tolerance or food consumption. A.** Week 14 plasma glucose levels during the ITT test after 4 hours of fasting in HFD mice (n=5,two-way ANOVA, F_3,87_ = 1.037, p=0.38). AUC graphs from 14-week ITT tests of HFD mice (n=5, one-way ANOVA, F _3,15_=0.189, p=0.902). **B.** Week 14 plasma glucose levels during the ITT test following 4 hours of fasting in chow-fed mice (n=5, two-way ANOVA, F _3,96_=112.1, ****p<0.0001) (*post hoc* comparison, N=4/5, Tukey’s multiple comparison tests, p<0.05). AUC graphs from 14-week ITT tests chow-fed mice (n=5, one-way ANOVA; F _3,16_=1.103, ****p<0.0001) (*post hoc* comparison, n=5; Tukey’s multiple comparison test, p<0.05). (*Compares No repeated Shock F vs No repeated Shock M, #Compares No repeated shock F to Repeated Shock M, +Compares Repeated shock F to No repeated Shock M, %Compares Repeated shock F to Repeated Shock M). **C.** Total food consumption of HFD-fed mice over 60 hours in the metabolic chamber (n=4/5; one-way ANOVA, p>0.05). **D.** Total food consumption of chow-fed mice in the metabolic chamber over 60 hours (n=5; one-way ANOVA, p>0.05).

**Supplemental Fig. 3. Sex and acute stress effects on mice that are fed chow/HFD. A.** Energy expenditure (EE) (kCal/hr) of HFD-fed female and male mice during 60 hours of metabolic chamber housing (n=5, One way ANOVA, p>0.05). **B.** Energy expenditure (EE) (kCal/hr) of chow-fed female and male mice during 60 hours of metabolic chamber housing (N=5, One way ANOVA, p>0.05) **C.** Respiratory exchange ratio (RER) in HFD-fed female and male mice (N=5, One way ANOVA, p>0.05) **D.** RER in chow-fed female and male (N=4,5, One way ANOVA, p>0.05) **E.** Pedestrian locomotion (m) in HFD-fed female and male mice (N=5, One way ANOVA, **p<0.01) (*Post hoc* comparison, Tukey’s multiple comparison test, p<0.05). **F.** Pedestrian locomotion (m) in chow-fed female and male mice (N=5, One way ANOVA, **p<0.01) (*Post hoc* comparison, Tukey’s multiple comparison test, p<0.05).

**
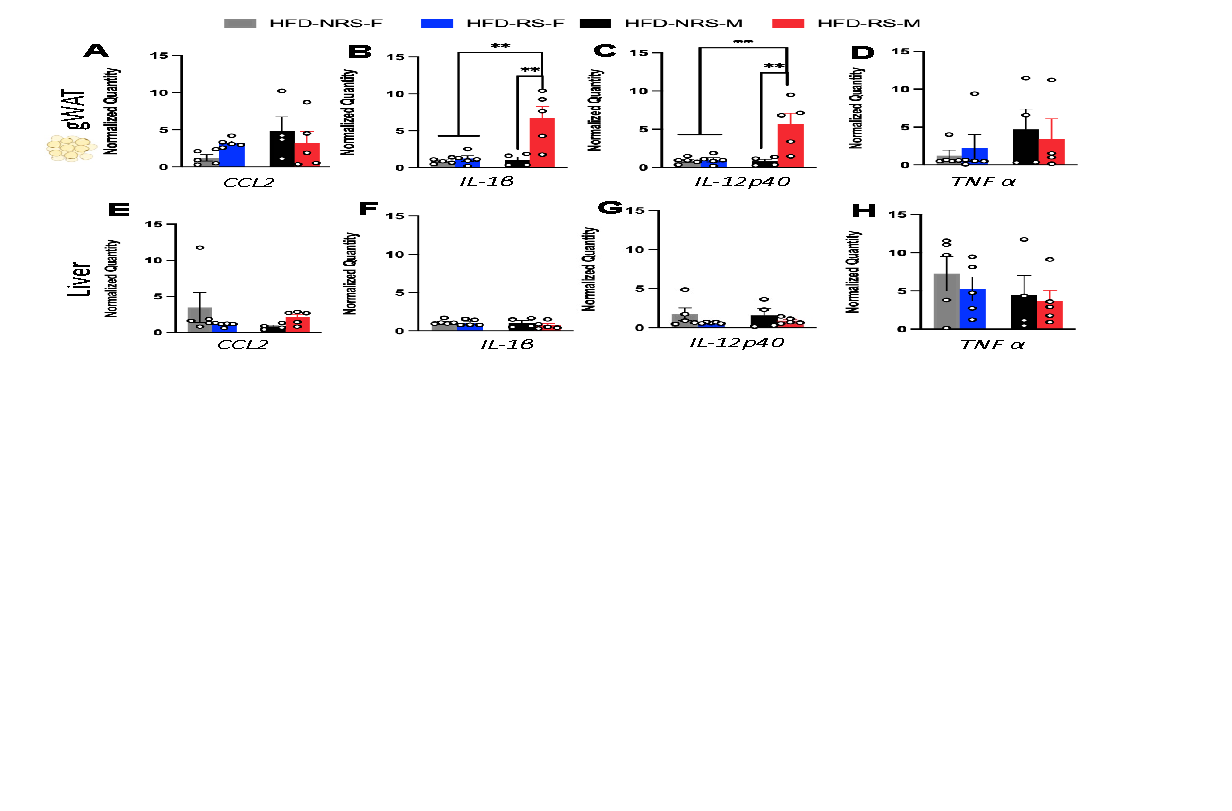
**

**Supplemental Fig 4.** **HFD induces upregulation of inflammatory markers in the peripheral tissue. A.** Normalized quantification of CCL2 gene expression in gWAT of HFD fed animals (n=9, two way ANOVA, F_1,15_  = 2.248, p=0.1545) **B.** Quantification of pro-inflammatory cytokine IL-1β in gWAT of HFD mice (N=9, two way ANOVA, F_1,15_  = 9.796 &11.08 respectively, **p<0.01) (*Post hoc* comparison, N=4/5, Tukey’s multiple comparison test, **p<0.01). **C.** Quantification of cytokine IL-12p40 in gWAT of HFD fed mice (n=9/10, two way ANOVA, F_1,15_  = 9.796 & 11.08 respectively, *p<0.05 & **p<0.01 respectively) (*Post hoc* comparison, n=4/5, Tukey’s multiple comparison test, **p<0.01). **D.** Quantification of TNF α levels in HFD fed animals in the gWAT(n=9, two way ANOVA, p=0.26). **E,F,G &H.** Quantification of CCL2, IL-1β, IL-12p40 or TNF α gene expression levels in liver of HFD-fed animals (N=9, two way ANOVA, p<0.05).


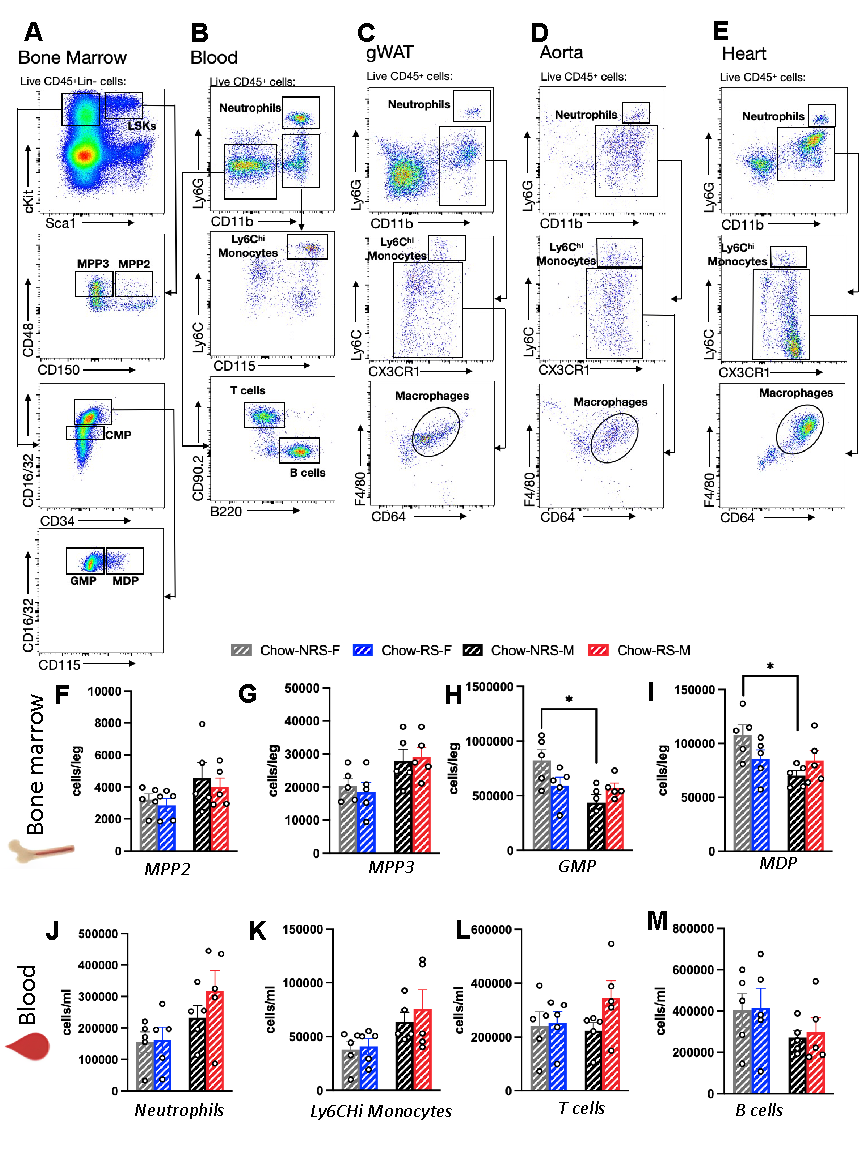


**Supplemental Fig. 5 Chow diet has minimal effects on peripheral myeloid lineage cells and inflammatory markers. A-E.** Gating strategies for flow cytometric data analysis. **F.** FACS analysis and further quantification of MPP2 cells in the BM of HFD (n=10, two way ANOVA, F_1,16_  = 4.007, p=0.06). **G.** Quantification of MPP3 cells in the BM of chow-fed animals (n=10, two way ANOVA, F_1,16_  = 9.504, p=0.07) **H.** Quantification of GMPs in BM of chow fed animals (n=10, two way ANOVA, F_1,16_  = 7.478, *p<0.05) (*Post hoc* comparison, n=5, Tukey’s multiple comparison test,* p<0.05). **I.** Quantification of MDPs in the BM of chow animals (N=10, two way ANOVA, F_1,16_  = 5.604, *p<0.05) (*Post hoc* comparison, N=5, Tukey’s multiple comparison test,* p<0.05). **J.** Quantification of neutrophil levels in the blood of chow fed mice (N=9, two way ANOVA, F_1,16_  = 6.46, p=0.02) (*Post hoc* comparison, N=5, Tukey’s multiple comparison test, p>0.05). **K.** Quantification of Ly6CHi monocytes in the blood of chow fed mice (n=10, two way ANOVA, F_1,16_  = 6.46, *p<0.05) (*Post hoc* comparison, N=5, Tukey’s multiple comparison test, p>0.05). **L.** Quantification of T cells in the blood of chow-fed animals (n=9, two way ANOVA, F_1,16_  = 0.581, p=0.45). **M.** Quantification of B cells in the blood of chow-fed animals (n=10, two way ANOVA, F_1,16_  = 2.77, p=0.16).


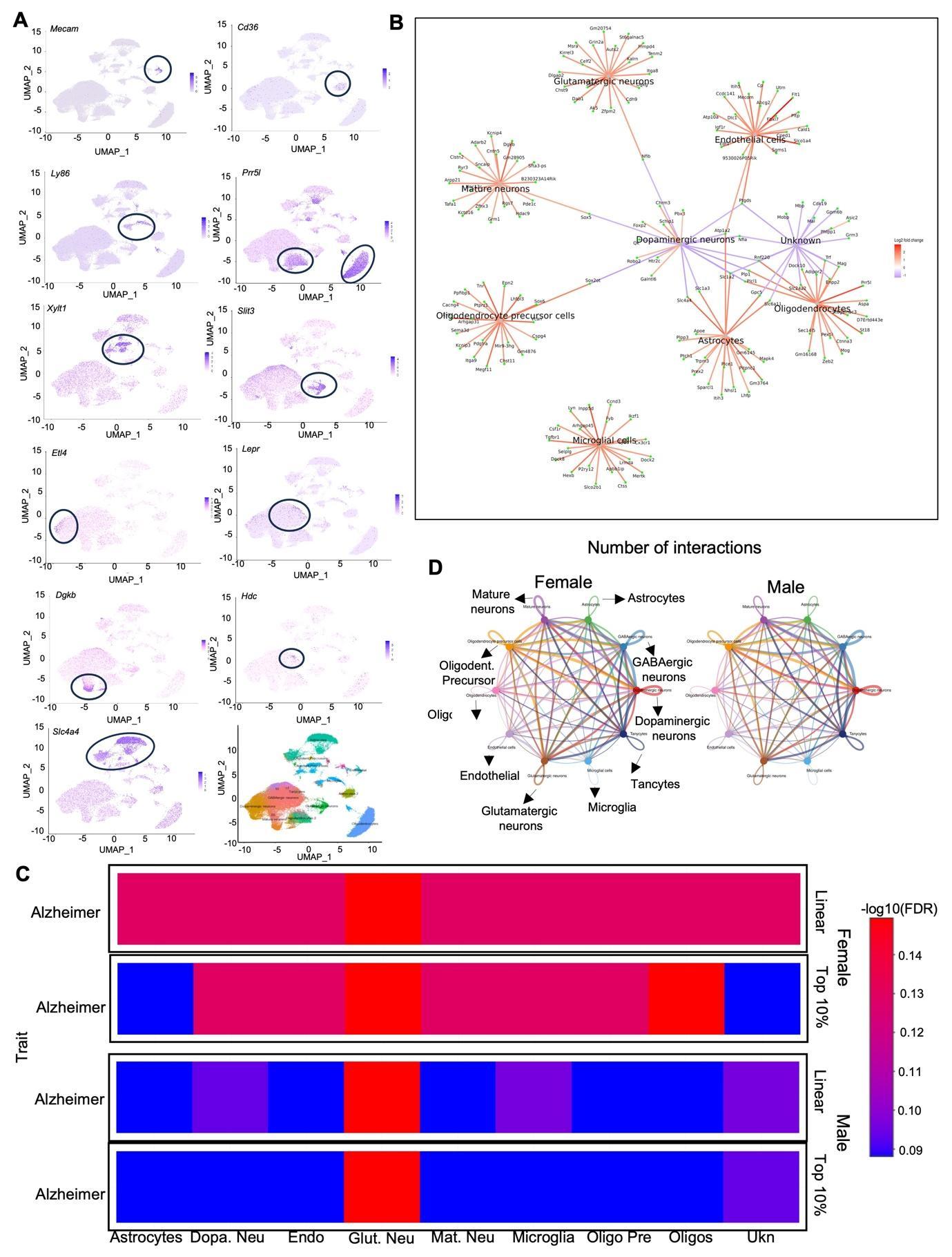


**Supplemental Fig. 6. scRNA-seq from VMH show celltype specific gene expression, cellular interaction and GWAS phenotype in males and females. A.** The UMAP plot depicts the distribution of annotated clusters from the combined dataset based on indicated marker genes. **B.** Analysis of cell-type-specific feature genes and shared gene connections within labeled clusters. **C.** A GWAS study reveals Alzheimer traits associated with differentially expressed genes (DEGs) in male and female mice across specified cell types. **D.** The interactome illustrates the number of interactions between cell types, derived from ligand-receptor interactions in males and females.
